# New factors in heart failure pathophysiology: Immunity cells release of extracellular vesicles

**DOI:** 10.3389/fcvm.2022.939625

**Published:** 2022-11-03

**Authors:** Alba Vilella-Figuerola, Teresa Padró, Eulàlia Roig, Sònia Mirabet, Lina Badimon

**Affiliations:** ^1^Cardiovascular-Program ICCC, IR-Hospital Santa Creu i Sant Pau, IIB-Sant Pau, Barcelona, Spain; ^2^Department of Biochemical and Molecular Biology, Universitat Autònoma de Barcelona, Barcelona, Spain; ^3^Centro de Investigación Biomédica en Red Cardiovascular, Instituto de Salud Carlos III, Madrid, Spain; ^4^Heart Failure Group, Department of Cardiology, Hospital Santa Creu i Sant Pau, Barcelona, Spain; ^5^UAB-Chair Cardiovascular Research, Barcelona, Spain

**Keywords:** extracellular microvesicles (EVs), chronic heart failure, immune cells, inflammation, liquid biopsies, extracellular vesicle (EV)

## Abstract

Leukocyte-shed extracellular vesicles (EVs) can play effector roles in the pathophysiological mechanisms of different diseases. These EVs released by membrane budding of leukocytes have been found in high amounts locally in inflamed tissues and in the circulation, indicating immunity cell activation. These EVs secreted by immune cell subsets have been minimally explored and deserve further investigation in many areas of disease. In this study we have investigated whether in heart failure there is innate and adaptive immune cell release of EVs. Patients with chronic heart failure (cHF) (*n* = 119) and in sex- and age-matched controls without this chronic condition (*n* = 60). Specifically, EVs were quantified and phenotypically characterized by flow cytometry and cell-specific monoclonal antibodies. We observed that even in well medically controlled cHF patients (with guideline-directed medical therapy) there are higher number of blood annexin-V^+^ (phosphatidylserine^+^)-EVs carrying activated immunity cell-epitopes in the circulation than in controls (*p* < 0.04 for all cell types). Particularly, EVs shed by monocytes and neutrophils (innate immunity) and by T-lymphocytes and natural-killer cells (adaptive immunity) are significantly higher in cHF patients. Additionally, EVs-shed by activated leukocytes/neutrophils (CD11b^+^, *p* = 0.006; CD29^+^/CD15^+^, *p* = 0.048), and T-lymphocytes (CD3^+^/CD45^+^, *p* < 0.02) were positively correlated with cHF disease severity (NYHA classification). Interestingly, cHF patients with ischemic etiology had the highest levels of EVs shed by lymphocytes and neutrophils (*p* < 0.045, all). In summary, in cHF patients there is a significant immune cell activation shown by high-release of EVs that is accentuated by clinical severity of cHF. These activated innate and adaptive immunity cell messengers may contribute by intercellular communication to the progression of the disease and to the common affectation of distant organs in heart failure (paracrine regulation) that contribute to the clinical deterioration of cHF patients.

## Introduction

Leukocyte-shed extracellular vesicles (EVs) can play effector roles in the pathophysiological mechanisms of different diseases. These EVs released by membrane budding of leukocytes have been found in high amounts locally in inflamed tissues and in the circulation, indicating immunity cell activation. These EVs secreted by immune cell subsets have been minimally explored and deserve further investigation in many areas of disease (1). Heart failure (HF) is a complex syndrome that accounts for a high proportion of cardiovascular death worldwide (2, 3). It is characterized by an impairment of the cardiac function due to genetic and environmental insults and comorbidities such as ischemic disease, that induce significant alterations of heart structure (left ventricle hypertrophy or dilation, fibrosis, maladaptive remodeling) and function (decreased cardiac output, increased end-diastolic pressure and diastolic or systolic dysfunction) (4). HF can be classified in HF with preserved ejection fraction (HFpEF), HF with reduced ejection fraction (HFrEF) and in HF with mildly reduced ejection fraction (HFmrEF), according to their echographic values of left ventricular ejection fraction (LVEF) (2, 5). Although these groups present common symptomatology, their pathophysiological mechanisms and therapeutic approaches are slightly different. In that sense, although this is not universal, the vast majority of HFrEF have underlying ischemic etiology, while HFpEF patients tend to develop HF due to hypertensive or heart valve disease (6, 7).

The systemic inflammation of these patients represents a chronic and non-resolving health impairment with the presence of elevated levels of circulating cytokines or pro-inflammatory mediators, such as TNFα, IL-β1, IL-6, or CRP (4, 8). Further to the raised levels of inflammatory biomarkers, another source of organ damage is the defective equilibrium between pro-inflammatory and anti-inflammatory immune-cell subsets, such as monocytes/macrophages, neutrophils, lymphocytes (B, Treg, Th), that often results in impaired repair processes (9–11). Several clinical trials have tried to target the on-going inflammation with neutral results (12, 13). The failure of these approaches suggests that the potentially involved pathways are not well understood, and that further research in the pathophysiology of the disease is needed. Indeed, HF is a spectrum of overlapping phenotypes that need to be more accurately classified.

Circulating extracellular microvesicles (EVs) are a heterogeneous population of extracellular vesicles with a size range between 20 and 1,000 nm. Composed of a phosphatidylserine-rich phospholipid bilayer that exposes transmembrane proteins and receptors from their parental cells, EVs are released after physiological stimuli such as cell activation, damage, stress, apoptotic or necrotic damage (14, 15). EVs play a role in physiology and pathophysiology since they are involved in various processes such as cell adhesion, communication (they carry miRNA, proteins and lipids), apoptosis, immune response, vascular function, remodeling, hemostasis or thrombosis (16). Their cargo reflects their parental cell condition and metabolic state and its numbers have been correlated with disease progression and severity (14, 16, 17). In cardiovascular disease, they have been related to several pathologies (18) including atherosclerosis (19–21), coronary artery disease (14), ischemia and myocardial infarction (22–24) and stroke (25).

To gain more insight in the pathophysiology of chronic HF (cHF), we aimed to investigate whether immune cells were activated in patients, even though treated according to guideline-directed medical therapy. Several studies have investigated endothelial-derived EVs in relation with cardiovascular risk factors and cardiovascular disease pathophysiology (26–29). However, to our knowledge, there are no studies investigating the type of immune cells that are activated and shed EVs in cHF.

In this study we have investigated whether in chronic heart failure there is innate and adaptive immune cell release of EVs and whether specific circulating EV patterns associate with the underlying cHF etiology (ischemic and non-ischemic) or the disease severity in cHF patients.

## Materials and methods

### Clinical study population

A total of 119 ambulatory patients with clinical diagnosis of cHF and under guideline-directed medical therapy were prospectively recruited in the outpatient HF unit of the Hospital de la Santa Creu i Sant Pau (Barcelona, Spain) between September 2016 and July 2018 and were followed-up until October 2019. During follow-up patients were treated according to international guidelines and at clinician discretion in the outpatient unit of our hospital. A control group was also included, comprising 60 sex- and age-matched controls without cHF. Baseline demographic data, classical cardiovascular risk factors and background medication of the study population (cHF and control groups) are shown in Table 1. Exclusion criteria were: HF with mildly reduced ejection fraction (40–50% of left ventricular ejection fraction [LVEF]), past history of cancer, inflammatory disorders, sepsis or infection. Pregnant women were also excluded. Characteristics of the cHF patients including biochemical and hematological data, cHF-etiology and cHF-severity (according to the New York Heart Association Classification [NYHA]) are given in Table 2 and Supplementary Tables 1, 2. NYHA classification system considers cardiac functionality and disease severity, dividing cHF in four categories (5). Briefly, NYHA I patients do not present limitations in their physical activity and daily activity does not cause breathlessness, fatigue or palpitations. NYHA II patients have a slight limitation of physical activity, and whereas they are comfortable at rest, ordinary activity produces breathlessness, fatigue or palpitations. Patients in NYHA III present a marked limitation of physical activity. Although they are comfortable at rest, less than ordinary physical activity results in breathlessness, fatigue or palpitations. Finally, NYHA IV patients are unable to carry on any physical activity without discomfort and can have symptoms at rest. In patients with ischemic etiology, ischemia was defined as history of a previous myocardial infarction, or in the absence of an acute ischemic event, if there was stenosis equal or greater than 75% in the common trunk or left anterior descending artery, as well as when stenosis was equal or greater than 75% in two or more of the principal coronary arteries.

**TABLE 1:** Patients and controls baseline characteristics.

	Controls *n* = 60	cHF *n* = 119	*P*-value controls-cHF
**Demographic characteristics; mean ± SD**			
Male/Female, n	34/26	81/38	0.133
Age, years	67.1 ± 7.1	67 ± 11.8	0.553
Systolic blood pressure, mmHg	144.8 ± 20.2	120.4 ± 19.1	**0.000**
Diastolic blood pressure, mmHg	83.7 ± 11.3	73.9 ± 11.1	**0.000**
Left ventricular ejection fraction,%	52–74^†^	45.59 ± 18.98	–
**Risk factors; n (%)**			
Smokers	10 (16)	13 (10.9)	0.278
Hypertension	33 (55)	82 (68.9)	0.067
Pulmonary hypertension	–	49 (41.1)	–
Diabetes mellitus	10 (16)	53 (44.5)	**0.000**
Dyslipidaemia	45 (75)	64 (53.7)	**0.004**
Chronic kidney disease	2 (3.3)	46 (38.6)	**0.000**
Atrial fibrillation	–	50 (42)	–
**Background medication; n (%)**			
Angiotensin-converting-enzyme inhibitors	19 (31.6)	48 (40.3)	0.333
Angiotensin II receptor blockers	9 (15)	35 (29.4)	0.054
Beta-blockers	2 (3.3)	100 (84)	**0.000**
Aldosterone antagonists	–	66 (55.4)	–
Diuretics^‡^	6 (10)	104 (87.3)	**0.000**
Angiotensin receptor neprilysin inhibitors	–	17 (14.2)	–
Ivabradine	–	14 (11.7)	–
Statins	37 (61.6)	77 (64.7)	0.690
Insulin	3 (5)	16 (13.4)	0.083
Anti-diabetic drugs	8 (13.3)	40 (33.6)	**0.004**
Antiplatelet agents	12 (20)	46 (38.6)	**0.012**
Anticoagulants	2 (3.3)	61 (51.2)	**0.000**
Anti-arrhythmic drugs	–	26 (21.8)	–

^†^LVEF normal range (excerpted from https://www.ncbi.nlm.nih.gov/books/NBK459131/ on 18/01/2021).

^‡^Includes: furosemide, hydrochlorothiazide, torasemide and indapamide.

SD, standard deviation; cHF, chronic heart failure. The statistically significant *p*-values are in bold.

**TABLE 2:** Clinical characteristics of cHF patients at baseline (*n* = 119).

Patients characteristics	
**Clinical history; n(%) cHF etiology**	
Ischemic	43 (36.1)
Non-ischemic	76 (63.9)
Hypertensive cardiomyopathy	19 (15.9)
Dilated cardiomyopathy	24 (20.1)
Hypertrophic cardiomyopathy	12 (10)
Heart valve disease	17 (14.2)
Other	4 (3.3)
**New York Heart Association cHF stage**	
NYHA I	0 (0)
NYHA II	52 (43.7)
NYHA III	66 (55.5)
NYHA IV	1 (0.8)
Hospitalizations in the 6 months prior study initiation	49 (41.1)
**Re-events in the year prior study initiation**	9 (7.6)
Percutaneous coronary intervention	4 (44.5)
Coronary artery bypass grafting	2 (22.2)
Medical treatment	3 (33.3)
**Biochemistry; mean ± SD**	
Hemoglobin, mg/dl	129.8 ± 18.5
Creatinine, mg/dl	1.3 ± 0.54
C-Reactive Protein, mg/ml	7.63 ± 11
NT-proBNP, pg/ml	2954.8 ± 4211.3
High-sensitive troponin T, ng/l	27.7 ± 20.9
Erythrocytes, 10^6^/mm^3^	3.98 ± 0.74
Platelets, 10^3^/mm^3^	173.2 ± 56.89
Leukocytes, mm^3^	7426.1 ± 1950
Neutrophils, 10^9^/L	4.74 ± 1.54
Monocytes, 10^9^/L	0.75 ± 0.54
**Major outcomes during follow-up; n (%)**	
**Cardiovascular event** ^†^	26 (21.8)
Stroke	8 (6.7)
Aortic dissection	1 (0.8)
AMI + Cardiogenic shock	3 (2.5)
HTx/HTx waiting list	10 (8.4)/1 (0.8)
CV death	7 (5.8)
Emergency hospital admission for cHF	16 (13.4)
Rehospitalisation for cHF	57 (47.8)
Aortic aneurism	1 (0.8)
Other death causes^‡^	10 (8.4)

^†^Includes patients that suffered a stroke, an aortic dissection, an AMI, a cardiogenic shock, a CV death (mainly due to cHF) or were admitted to the emergency department. It does not include patients that underwent a HTx.

^‡^Includes patients that died due to a septic shock, a hemorrhage or a non-successful HTx. AMI, acute myocardial infarction; CV, cardiovascular; cHF, chronic heart failure; HTx, heart transplantation; NT-proBNP, N-terminal pro-hormone of brain natriuretic peptide; NYHA, New York Heart Association; SD, standard deviation.

The ethics committee at the Hospital de la Santa Creu i Sant Pau in Barcelona (Spain) approved the study (Ref 16/44) and it was conducted under the principles of the Declaration of Helsinki. A written informed consent was obtained from all participants prior recruitment.

### Blood sampling, circulating extracellular vesicles isolation and characterization

Venous blood was withdrawn from the cubital vein without tourniquet using a 20-gauge needle after 10–14 h of fasting into 3.8% sodium citrate tubes (BD Vacutainer, Becton Dickinson). All samples were processed identically and within the first 2 h. Blood was centrifuged at 1,560 g for 20 min at 20°C (Eppendorf 5810R GLOOB04932 centrifuge, A-4-81 rotor, Eppendorf) to avoid *in vitro* platelet activation. Platelet-poor plasma (PPP) was carefully aspirated, leaving about a 1 mm undisturbed layer on top of cells. A second centrifugation step was then performed at 1,500 g for 10 min at 20°C (Eppendorf 5415R centrifuge, FA45-24-11 rotor, Eppendorf) to ensure the complete removal of cells and obtain the platelet-free plasma (PFP). PFP aliquots were stored at −80°C until flow cytometry studies.

The EVs fraction was isolated from PFP by a two-step high-speed centrifugation, according to the procedure previously described (20, 24, 30). Specifically, PFP was thawed and centrifuged at 1,500 g for 10 min at 20°C (Eppendorf 5417R centrifuge, FA45-24-11 rotor, Eppendorf). Next, 250 μl of PFP were collected from the upper part of the vial and transferred to a new tube to pellet the EVs, and then centrifuged at 20,000 g for 30 min at 20°C (Eppendorf 5417R centrifuge, FA45-24-11 rotor, Eppendorf). The supernatant (225 μl) was discarded, and the EVs-enriched pellet was washed with 225 μl of citrate-phosphate buffered saline (PBS) solution (citrate-PBS; 1.4 mmol/L phosphate, 154 mmol/L NaCl, 10.9 mmol/L trisodium citrate, pH 7.4) before a second equal centrifugation (20,000 g, 30 min, 20°C) was pursued. Finally, the remaining pellets were resuspended in a final volume of 100 μl citrate-PBS.

### Flow cytometric analysis of extracellular vesicles

Extracellular vesicles were phenotypically characterized by three-label flow cytometric analysis, performed as described previously (20, 24, 30). Washed EVs suspensions were diluted in PBS containing 2.5 mmol of CaCl_2_ (Annexin binding buffer [ABB], BD Biosciences, San Jose). Afterward, combinations of CFBlue-conjugated annexin V (AV) (Immunostep, Salamanca, Spain) to detect phosphatidylserine, and two specific monoclonal antibodies (mAb) (Supplementary Table 3) labeled with fluorescein isothiocyanate (FITC) and phycoerythrin (PE), or the isotype-matched control mAb, were added. Samples were incubated for 20 min at 20°C in the dark and diluted with ABB before being immediately analyzed on a FACSCantoII™ (Becton Dickinson, Franklin Lakes, NJ, USA) flow cytometer.

Sample acquisition was performed during 1 min per sample at “low flow” rate. Forward scatter (FSC), side scatter (SSC), and fluorescence data were obtained with the settings in the logarithmic scale. Gate limits were established as follows with the same criteria previously described (20, 24, 30). The upper threshold for FSC was set with the Megamix-Plus FSC beads (BioCytex, Marseille, France). Megamix-Plus FSC beads for cytometer setting are a mix of beads of the following bead-equivalent diameters: 0.1, 0.3, 0.5, and 0.9 μm. According to the beads signal, the lower detection limit was placed as a threshold above the electronic background noise of the flow cytometer for FSC and the second logarithm for SSC. EVs within the established gate limits (>0.1 to 1 μm) were identified and quantified based on their binding to AV and reactivity to cell-specific mAb. To identify positive marked events, thresholds of fluorescence were also set based on samples incubated with the same final concentration of isotype-matched control mAb after titration experiments. AV binding level was corrected for auto-fluorescence using fluorescence signals obtained with EVs in a calcium-free buffer (PBS). Additional controls to correct for FITC-, PE- and CFBlue-fluorescence were also pursued (unstained and single-stained controls), as well as serial dilutions to ensure proper event detection and to prevent swarming. Finally, to corroborate the presence of EVs in the EVs suspension, 5% saponin-treated controls were performed (see Supplementary Figure 1). To reduce background noise, buffers were prepared on the same day and filtered through 0.22 μm pore-size filters under vacuum. EVs markers observed in cHF patients by flow cytometry were validated by western blot as described in Supplementary material and shown in Supplementary Figure 2.

Data was analyzed with BD FACSDiva™ Software (version 6.1.3, Becton Dickinson, Franklin Lakes, NJ, USA). The concentration (number of EVs per μl of plasma) was determined according to Nieuwland’s procedure (31), based on sample’s volume, flow cytometer’s flow rate and the number of fluorescence-positive events (N), as follows: EVs/μl = N x (Vf/Va) x (Vt/FR) x (1/Vi), where Vf(μl) = final volume of washed EVs suspension, Va(μl) = volume of washed EVs suspension used for each labeling analysis, Vt(μl) = volume of EVs suspension before fluorescence-activated cell sorting analysis, FR(μl/min) = flow rate of the cytometer at low mode (the average volume of EVs suspension analyzed in 1 min), 1 is the μl unit of volume, and Vi(μl) = original volume of plasma used for EVs isolation.

### Statistical analysis

Statistical analysis was performed using SPSS Statistical Analysis System (version 26.0, IBM Corp. Armonk, NY). Normality of variables was assessed with Saphiro–Wilks test. Descriptive analysis for qualitative variables was performed using number of cases and percentages, while for quantitative variables, mean ± SD were used except when specified. Frequencies of qualitative variables (clinical outcomes, risk factors, and medications) were compared between groups using Chi-squared analysis. Median values of quantitative variables were contrasted with non-parametric tests. Statistical significances between groups were determined with U Mann–Whitney tests. Correlation analyses were pursued using the Spearman correlation test. Receiver operating characteristic (ROC) curve analyses for predicted probabilities were performed to identify threshold concentrations of EVs able to discriminate between cHF NYHA stages, or between severity in ischemic patients, and the corresponding area under the curve (AUC) with its 95% confidence interval (CI) was calculated. Binary logistic regression models were pursued to estimate predicted probabilities for NYHA severity using combinations of EVs and/or the currently used biomarker NT-proBNP. A *p* < 0.05 was considered statistically significant. Sample size was determined using the GRANMO sample size calculator (version 7.12, April 2012). To detect mean differences in the number of EVs, a total of 126 subjects (controls and cHF) would be needed to complete the study (α risk = 0.05, beta risk = 0.2, two-sided test).

## Results

### Clinical characteristics of the study population

Mean age of cHF was 67 ± 12 years (68% of men). Sixty cHF patients presented reduced ejection fraction (HFrEF; LVEF < 40%; *n* = 60) while 59 (49.6%) had cHF with preserved ejection fraction (HFpEF; LVEF > 50%). Additionally, 52 (43.7%) cHF presented a less severe cHF symptomatology according to the NYHA classification (class II), while 66 (55.5%) and 1 (0.8%) were in the higher severity spectrum, being in the NYHA stages III and IV, respectively. Demographics, classical risk factors and background medication are shown in Table 1, while etiology, biochemistry data and follow-up events and outcomes are listed in Table 2. Demographical, pharmacological and classical risk factor data of cHF according to their ejection fraction or severity (NYHA classification) is disclosed in Supplementary Tables 1, 7. Underlying etiology classification and clinical data of cHF according to these groups are listed in Supplementary Tables 2, 8.

### Immunity cell-derived circulating extracellular vesicles: Cell-origin and activation

**FIGURE 1:**
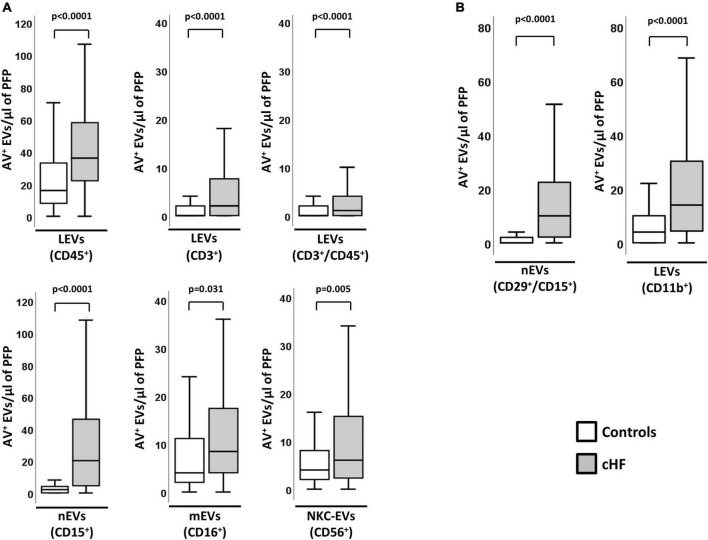
Cellular origin and distribution of AV^+^ EVs in cHF and controls. **(A)** Distribution of EVs from total leukocytes (CD45^+^), T-lymphocytes (CD3^+^ and CD3^+^/CD45^+^), neutrophils (CD15^+^), monocyte/macrophage (CD16^+^) and natural-killer cells (CD56^+^). **(B)** Distribution of EVs from activated neutrophils (CD29^+^/CD15^+^) and from activated leukocytes (CD11b^+^). A *p* < 0.05 was considered significant (U Mann–Whitney test). AV^+^, annexin V^+^; cHF, chronic heart failure; EVs, extracellular microvesicles; LEVs, leukocyte-derived EVs; lEVs, lymphocyte-derived EVs; mEVs, monocyte-derived EVs; nEVs, neutrophil-derived EVs; NKC-EVs, natural-killer cells-derived EVs; PFP, platelet-free plasma.

Total levels of EVs-AV^+^ shed by leukocytes were significantly higher in cHF patients than in controls. As shown in Figure 1A, EVs carrying the pan-leukocyte marker CD45^+^, the T-lymphocyte co-receptor (CD3^+^) or both (CD3^+^/CD45^+^), were significantly increased in cHF (*p* < 0.0001). In addition, patients had significantly higher amounts of monocyte- and neutrophil-derived AV^+^-EVs carrying CD16^+^ (receptor Fcγ III) and CD15^+^ (sialyl Lewis X), respectively. EVs shed by natural-killer cells carrying the neural cell adhesion molecule-1 (CD56^+^) were significantly elevated in cHF compared to controls.

In addition, cHF patients had significantly higher levels of EVs derived from activated immune cells (identified for the shedding of activation markers in EVs). Specifically, AV^+^-EVs from activated leukocytes (integrin α-M; CD11b^+^) and activated neutrophils (integrin β-1/sialyl Lewis X; CD29^+^/CD15^+^) were significantly increased in patients compared to controls (Figure 1B).

The activation of the immunity cells measured by EVs did not correlate with the number of blood leukocytes or CRP levels, indicating different pathophysiological pathways. Interestingly, circulating EVs from T-lymphocytes (CD3^+^/CD45^+^/AV^+^) significantly correlated with the cardiac damage markers hsTnT and NT-proBNP, while levels of EVs from activated neutrophils (CD29^+^/CD15^+^/AV^+^) and hsTnT and NT-proBNP showed a trend toward significance (Supplementary Table 4). cHF and control groups did not differ in the percentages of EVs shed by platelets (CD41a^+^; αIIbβ3-integrin).

### Immune cell-derived circulating extracellular vesicles signature and chronic heart failure severity

**FIGURE 2:**
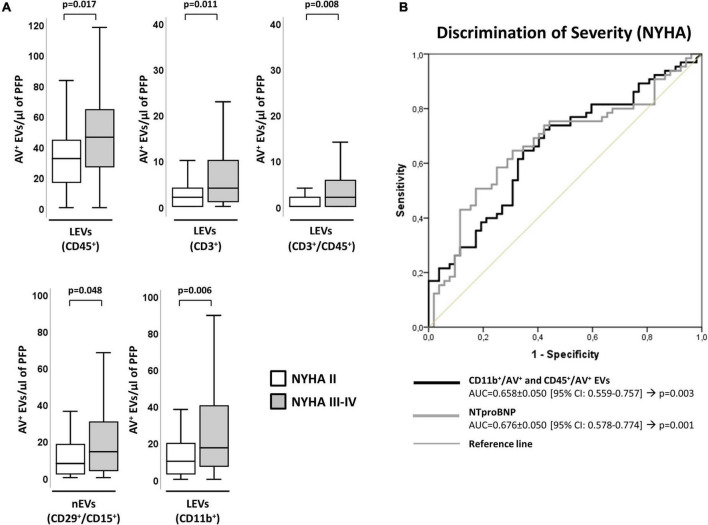
Distribution of AV^+^-EVs of immune-cell origin considering disease severity according to NYHA. **(A)** Distribution of EVs from total leukocytes (CD45^+^), T-lymphocytes (CD3^+^ and CD3^+^/CD45^+^), activated leukocytes (CD11b^+^) and from activated neutrophils (CD29^+^/CD15^+^). A *p* < 0.05 was considered significant (U Mann–Whitney test). **(B)** ROC curve analyses to evaluate EVs and NT-proBNP association to NYHA severity, with AUC indicated along its 95% CI. A *p* < 0.05 was considered significant. AUC, area under the curve; AV^+^, annexin V^+^; cHF, chronic heart failure; CI, confidence interval; EVs, extracellular microvesicles; LEVs, leukocyte-derived EVs; lEVs, lymphocyte-derived EVs; nEVs, neutrophil-derived EVs; NYHA, New York Heart Association; PFP, platelet-free plasma; ROC, receiver operating characteristic curve.

Chronic heart failure patients with more severe form of disease (a higher score in the NYHA classification [NYHA III-IV]) had higher values of EVs derived from leukocytes (CD45^+^/AV^+^) and specifically those shed by T-lymphocytes (CD3^+^/AV^+^, CD3^+^/CD45^+^/AV^+^). In addition, a significant increase in EVs from activated leukocytes (CD11b^+^/AV^+^) and activated neutrophils (CD29^+^/CD15^+^/AV^+^) was observed in these patients, compared to those with NYHA II (Figure 2A). Interestingly, these differences were maintained when both groups were compared with controls (Supplementary Table 5).

Distribution of the cHF population according to disease severity (according to the NYHA classification) is given in Table 2. Patients’ characteristics according to degree of severity (NYHA II and NYHA III-IV stages) are shown in Supplementary Tables 1, 2. Disease severity score according to NYHA correlated positively with hsTnT (rho = 0.200; *p* = 0.031) and NT-proBNP (rho = 0.313; *p* = 0.001).

Leukocyte-derived EVs subtypes showed a significant association to NYHA severity, evidencing their involvement in disease pathophysiology. By C-statistics analysis, the clustering of CD11b^+^/AV^+^ and CD45^+^/AV^+^ showed an AUC (AUC = 0.658 ± 0.050 [95% CI: 0.559–0.757]; *p* = 0.003) (Supplementary Table 6) similar to NT-proBNP (AUC = 0.676 ± 0.050 [95% CI: 0.578–0.774]; *p* = 0.001), hormone secreted by cardiomyocytes in the heart ventricles in response to stretching caused by increased ventricular blood volume that signals for a different pathophysiological pathway to that mapped by EVs (Figure 2B and Supplementary Table 6).

Interestingly, no differences in immune cell-derived EVs levels/phenotypes were detected in HFpEF and HFrEF patients. Instead, as expected, a negative correlation of NT-proBNP and LVEF was observed (rho = −0.222, *p* = 0.015). These results indicate that immune cell activation is a common feature in patients with both HFpEF and HFrEF. Demographic, biochemical and clinical classification of the patients according to their LVEF is shown in Supplementary Tables 7–9.

### Circulating extracellular vesicles and chronic heart failure etiology: Ischemic and non-ischemic disease

**FIGURE 3:**
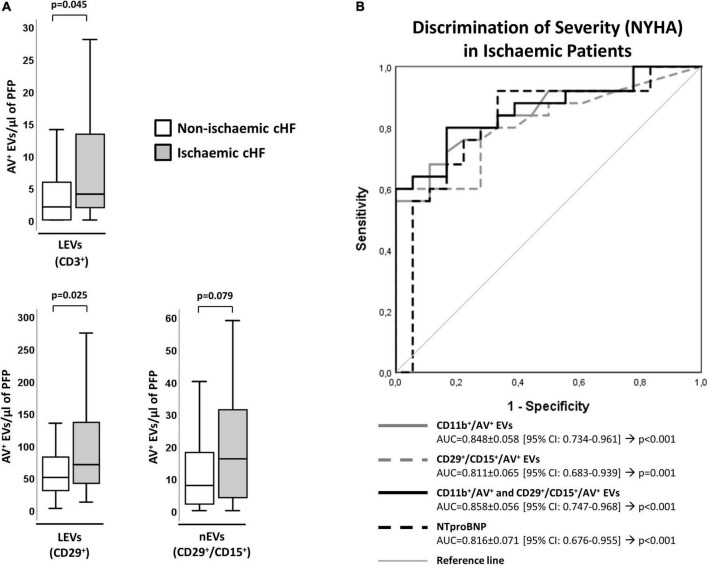
Distribution of AV^+^-EVs from immune-cell origin considering disease etiology. **(A)** Distribution of EVs from T-lymphocytes (CD3^+^), activated leukocytes (CD29^+^) and from activated neutrophils (CD29^+^/CD15^+^). A *p* < 0.05 was considered significant (U Mann–Whitney test). **(B)** ROC curve analyses were used to evaluate the impact of EVs and combinations of EVs with NT-proBNP on HF severity (NYHA) in ischemic patients (AUC indicated along its 95% CI). A *p* < 0.05 was considered significant. AUC, area under the curve; AV^+^, annexin V^+^; cHF, chronic heart failure; CI, confidence interval; EVs, extracellular microvesicles; LEVs, leukocyte-derived EVs; lEVs, lymphocyte-derived EVs; nEVs, neutrophil-derived EVs; NYHA, New York Heart Association; PFP, platelet-free plasma; ROC, receiver operating characteristic curve.

Chronic heart failure patients with ischemic etiology (HF-Isch) presented increased levels of EVs. Specifically, EVs derived from T-lymphocytes (CD3^+^/AV^+^, *p* = 0.045) or carrying the leukocyte activation marker integrin β-1 (CD29^+^/AV^+^, *p* = 0.025) were significantly increased in HF-Isch compared to non-ischemic patients (HF-NIsch). Moreover, EVs from activated neutrophils (CD29^+^/CD15^+^/AV^+^) displayed higher levels in HF-Isch compared to HF-NIsch, although this trend was non-significant (*p* = 0.079) (Figure 3A).

**FIGURE 4:**
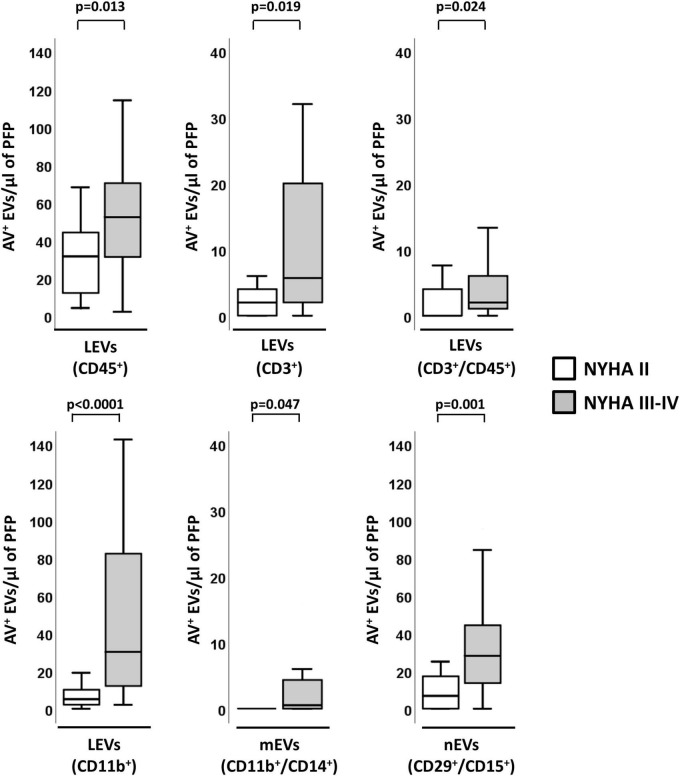
Distribution of AV^+^-EVs of immune-cell origin considering disease severity in ischemic patients. Distribution of EVs of total leukocytes (CD45^+^), T-lymphocytes (CD3^+^ and CD3^+^/CD45^+^), activated leukocytes (CD11b^+^) and from activated monocytes (CD11b^+^/CD14^+^) and activated neutrophils (CD29^+^/CD15^+^). A *p* < 0.05 was considered significant (U Mann–Whitney test). AV^+^, annexin V^+^; EVs, extracellular microvesicles; LEVs, leukocyte-derived EVs; lEVs, lymphocyte-derived EVs; mEVs, monocyte-derived EVs; nEVs, neutrophil-derived EVs; NYHA, New York Heart Association; PFP, platelet-free plasma.

Within HF-Isch, different EVs profiles associated to disease severity (NYHA class). There were significantly higher levels of EVs derived from leukocytes (CD45^+^/AV^+^; *p* = 0.013), T-lymphocytes (CD3^+^/AV^+^ and CD45^+^/CD3^+^/AV^+^; *p* < 0.024), and activated leukocytes, monocytes and neutrophils (CD11b^+^/AV^+^, CD11b^+^/CD14^+^/AV^+^ and CD29^+^/CD15^+^/AV^+^, respectively; *p* < 0.05) in severe disease patients. No differences associated to severity were detected in HF-NIsch (Figure 4). C-statistics analyses to assess the involvement of EVs in the pathophysiology and severity of disease in HF-Isch, showed that activated leukocytes (CD11b^+^/AV^+^) had the best discrimination power, with an AUC of 0.848 ± 0.058 (95% CI: 0.734–0.961; *p* < 0.001), followed by activated neutrophils (CD29^+^/CD15^+^/AV^+^), with and AUC of 0.811 ± 0.065 (95% CI: 0.683–0.939; *p* = 0.001). Again, in an order of magnitude comparable to the contribution of disease showed by NT-proBNP, with an AUC of 0.816 + 0.071 (95% CI: 0.676–0.955; *p* < 0.001). When analyses were performed with the combined probabilities of EVs from activated leukocytes and neutrophils (CD11b^+^/AV^+^ and CD29^+^/CD15^+^/AV^+^) the AUC increased to 0.858 ± 0.056 (95% CI: 0.747–0.968; *p* < 0.001), with a sensitivity and specificity of 80.0 and 83.3%, respectively. The addition of NT-proBNP to the analysis did not improve discrimination (AUC = 0.858 ± 0.056 [0.747–0.968]; *p* < 0.001) (Figure 3B and Supplementary Table 10) indicating two types of contribution to disease progression of EVs and NT-proBNP, respectively.

Extracellular vesicles shedding levels, considering elapsed time between any ischemic event and sample collection, was similar in those patients with longer elapsed time between ischemic event and blood withdrawal (more than 1 year: 7 ± 7 years) and those with lower time-periods (less than a year: 5 ± 2 months) (Supplementary Table 11).

## Discussion

Heart failure is a complex syndrome where the heart is unable to maintain a correct blood supply to ensure that the metabolic needs of the body are met (2). This can occur due to structural and functional defects, which at the same time are the result of major pathogenic mechanisms like hemodynamic overload, ischemia-related dysfunction, or ventricular remodeling among others (32). cHF patients have shown in diverse studies to have a chronic and non-resolving inflammatory state (11, 33).

The immune system has an important role in the setting of structural and functional changes in many organs, especially when the immune reparative processes become impaired. The present study has aimed at obtaining information on the activation state of different immunity cells in cHF patients. We have demonstrated that cHF patients present differential EVs profiles in comparison to control subjects, observing increased levels of EVs derived from leukocytes in cHF, reflecting the chronic activation state of these cells.

We have specifically detected EVs derived from lymphocytes, neutrophils, monocytes/macrophages and natural-killer cells in cHF compared to controls. In addition, we have shown that numbers of these EVs, except those shed by natural-killer cells and monocytes, correlated with the severity of the symptomatology. The combination of EVs CD11b^+^/AV^+^ and CD45^+^/AV^+^ was able to discriminate between NYHA stages II and III-IV, with an AUC of 0.658, indicating the implication of the activation of these immunity cells in disease severity pathophysiology. We also found that EVs could discriminate the etiology of cHF, with those patients with underlying ischemic etiology presenting significantly increased immune cell shed EVs. Further, we observed that in these patients EVs from activated leukocytes in combination with those from activated neutrophils, had a high discriminative potential in assessing cHF severity similar to NT-proBNP that measures another component of disease pathophysiology. We did not observe, however, differences in immune-cell-derived EVs profiles depending on the LVEF of cHF patients, indicating that both HFpEF and HFrEF patients have activation of immunity cells.

Several studies have shown how EVs can signal pathophysiological pathways of cardiovascular disease progression. For instance, our group has recently demonstrated that EVs can predict the presence of atherosclerotic plaques and discriminate plaque composition in familial hypercholesterolemia (21, 34). Further, it is widely accepted that EVs can identify severity of various pathologies (30, 35) and predict cardiovascular events (24, 36, 37), including cardiovascular death (22, 30). In fact, some studies have already observed increased levels of EVs in cHF, such as EVs from endothelial cells carrying the marker CD144^+^ (29).

Here, we have shown in patients under guideline-directed medical therapy that immunity cells are activated. The amount of EVs shed by neutrophils (CD29^+^/CD15^+^/AV^+^), T-lymphocytes (CD3^+^/AV^+^ and CD3^+^/CD45^+^/AV^+^) or carrying the activation marker CD11b^+^/AV^+^, positively correlates with disease severity. Further, we have shown for the first time that immune-cell-derived EVs associate to NYHA disease stage, and that immune cells are similarly activated in HFpEF and HFrEF patients. A non-resolving inflammation is a reported characteristic of HF (11, 38); in addition, our results indicate that there is an activated state of the innate and adaptive immune cells that may need be the target of future therapeutic interventions.

Patients with cHF of ischemic origin have significantly higher numbers of EVs from T-lymphocytes and neutrophils. This is a pathophysiological indication of the increase in neutrophils in the myocardium after an initial insult (10). Neutrophils are of the first cells mobilized to the damaged myocardium, where they clear diseased cells, start the reparative macrophages (M1) induction and promote inflammation, tissue reparation and fibrosis (10, 11, 39). Following the neutrophil and macrophage wave, T-lymphocytes infiltrate the heart and promote wound healing. However, if this pro-inflammatory state is prolonged in time and is not switched to a more anti-inflammatory phenotype, maladaptive remodeling and fibrosis occur, promoting HF (11, 39). This crosstalk among cells in the damaged myocardium is mapped by the phenotypically characterized EVs.

As study limitations we should first mention that the relatively small cohort size included in the study has impeded the adjustment for comorbidities and medications; however, this is a hypothesis generating study. Second, by experimental design, we did not include patients with HF with mildly reduced ejection fraction, which could have provided additional insights in the pathophysiology of the disease. Third, it is also worth mentioning that the CD11b marker (integrin α-M) is expressed by activated cells and indicates the type of activated cell when is associated with another CD marker. Additionally, due to methodological issues we could not analyze EVs released by B lymphocytes, nor determine cell counts of all white blood cells analyzed. However, in view of our results, further studies addressing EVs from B cells are warranted.

## Conclusion

In conclusion, HF is a complex syndrome where multiple pathophysiological pathways converge. In well treated chronic heart failure patients, immunity cells, both innate and adaptive immunity cells, are activated and release high number of circulating EVs. The released EVs may amplify the underlying inflammatory processes in these patients, affecting different tissues. Indeed, EVs are involved in crosstalk with other cells, acting as distant cell function regulators of targeted-receptor cells, even in distant organs.

## Data availability statement

The data that support the findings of this study are available from the corresponding author upon reasonable request.

## Ethics statement

The studies involving human participants were reviewed and approved by Ref 16/44; Ethics Committee of the Hospital de la Santa Creu i Sant Pau in Barcelona. The patients/participants provided their written informed consent to participate in this study.

## Author contributions

LB, TP, ER, and SM designed the research. AV-F performed the experiments. AV-F, TP, and LB analyzed the results. AV-F, TP, SM, and LB wrote and revised the manuscript. All authors contributed to the article and approved the submitted version.

## Abbreviations

AV: annexin V
cHF: chronic heart failure
EVs: extracellular microvesicles
HF-Isch: heart failure with an ischemic underlying etiology
HF-Nisch: heart failure with a non-ischemic underlying etiology
HFpEF: heart failure with preserved ejection fraction
HFrEF: heart failure with reduced ejection fraction
LVEF: left ventricle ejection fraction
NYHA: New York Heart Association.

## References

[1] Ridger VC Boulanger CM Angelillo-Scherrer A Badimon L Blanc-Brude OP Bochaton-Piallat M-L Microvesicles in vascular homeostasis and diseases position paper of the european society of cardiology (ESC) working group on atherosclerosis and vascular biology. *Thromb Haemost.* (2017) 117:1296–316. 10.1160/TH16-12-0943 28569921

[2] Kemp CD Conte JV. The pathophysiology of heart failure. *Cardiovasc Pathol.* (2012) 21:365–71. 10.1016/j.carpath.2011.11.007 22227365

[3] Virani SS Alonso A Benjamin EJ Bittencourt MS Callaway CW Carson AP Heart disease and stroke statistics—2020 update: a report from the American Heart Association. *Circulation.* (2020) 141:e139–596. 10.1161/CIR.0000000000000746 31992061

[4] Tanai E Frantz S. Pathophysiology of heart failure. *Compr Physiol.* (2016) 6:187–214. 10.1002/cphy.c140055 26756631

[5] McDonagh TA Metra M Adamo M Gardner RS Baumbach A Böhm M 2021 ESC Guidelines for the diagnosis and treatment of acute and chronic heart failure. *Eur Heart J.* (2021) 42:3599–726. 34447992 10.1093/eurheartj/ehab368

[6] Campbell RT McMurray JJV. Comorbidities and differential diagnosis in heart failure with preserved ejection fraction. *Heart Fail Clin.* (2014) 10:481–501. 10.1016/j.hfc.2014.04.009 24975911

[7] Lee DS Gona P Vasan RS Larson MG Benjamin EJ Wang TJ Relation of disease pathogenesis and risk factors to heart failure with preserved or reduced ejection fraction: insights from the framingham heart study of the national heart, lung, and blood institute. *Circulation.* (2009) 119:3070–7. 10.1161/CIRCULATIONAHA.108.815944 19506115 PMC2775498

[8] Testa M Yeh M Lee P Fanelli R Loperfido F Berman JW Circulating levels of cytokines and their endogenous modulators in patients with mild to severe congestive heart failure due to coronary artery disease or hypertension. *J Am Coll Cardiol.* (1996) 28:964–71. 10.1016/S0735-1097(96)00268-9 8837575

[9] Alvarez PA Briasoulis A. Immune modulation in heart failure: the promise of novel biologics. *Curr Treat Options Cardiovasc Med.* (2018) 20:26. 10.1007/s11936-018-0617-z 29541873

[10] Kain V Halade GV. Role of neutrophils in ischemic heart failure. *Physiol Genomics.* (2020) 205:107424. 10.1016/j.pharmthera.2019.107424 31629005 PMC6981275

[11] Shirazi LF Bissett J Romeo F Mehta JL. Role of inflammation in heart failure. *Curr Atheroscler Rep.* (2017) 19:27. 10.1007/s11883-017-0660-3 28432635

[12] Chung ES Packer M Lo KH Fasanmade AA Willerson JT. Randomized, double-blind, placebo-controlled, pilot trial of infliximab, a chimeric monoclonal antibody to tumor necrosis factor-α, in patients with moderate-to-severe heart failure: results of the anti-TNF therapy against congestive heart failure (ATTACH) trial. *Circulation.* (2003) 107:3133–40. 10.1161/01.CIR.0000077913.60364.D2 12796126

[13] Mann DL McMurray JJV Packer M Swedberg K Borer JS Colucci WS Targeted anticytokine therapy in patients with chronic heart failure: results of the randomized etanercept worldwide evaluation (RENEWAL). *Circulation.* (2004) 109:1594–602. 10.1161/01.CIR.0000124490.27666.B2 15023878

[14] Boulanger CM Loyer X Rautou P-E Amabile N. Extracellular vesicles in coronary artery disease. *Nat Rev Cardiol.* (2017) 14:259–72. 10.1038/nrcardio.2017.7 28150804

[15] Morel O Jesel L Freyssinet J-M Toti F. Cellular mechanisms underlying the formation of circulating microparticles. *Arterioscler Thromb Vasc Biol.* (2011) 31:15–26. 10.1161/ATVBAHA.109.200956 21160064

[16] Suades R Padró T Badimon L. The role of blood-borne microparticles in inflammation and hemostasis. *Semin Thromb Hemost.* (2015) 41:590–606. 10.1055/s-0035-1556591 26276937

[17] Badimon L Suades R Fuentes E Palomo I Padró T. Role of platelet-derived microvesicles as crosstalk mediators in atherothrombosis and future pharmacology targets: a link between inflammation, atherosclerosis, and thrombosis. *Front Pharmacol.* (2016) 7:293. 10.3389/fphar.2016.00293 27630570 PMC5005978

[18] Badimon L Suades R Vilella-Figuerola A Crespo J Vilahur G Escate R Liquid biopsies: microvesicles in cardiovascular disease. *Antioxid Redox Signal.* (2020) 33:645–62. 10.1089/ars.2019.7922 31696726

[19] Leroyer AS Isobe H Lesèche G Castier Y Wassef M Mallat Z Cellular origins and thrombogenic activity of microparticles isolated from human atherosclerotic plaques. *J Am Coll Cardiol.* (2007) 49:772–7. 10.1016/j.jacc.2006.10.053 17306706

[20] Suades R Padró T Vilahur G Badimon L. Circulating and platelet-derived microparticles in human blood enhance thrombosis on atherosclerotic plaques. *Thromb Haemost.* (2012) 108:1208–19. 10.1160/TH12-07-0486 23138460

[21] Suades R Padró T Alonso R Mata P Badimon L. High levels of TSP1+/CD142+ platelet-derived microparticles characterise young patients with high cardiovascular risk and subclinical atherosclerosis. *Thromb Haemost.* (2015) 114:1310–21. 10.1160/TH15-04-0325 26178021

[22] Chiva-Blanch G Bratseth V Ritschel V Andersen G Halvorsen S Eritsland J Monocyte-derived circulating microparticles (CD14+, CD14+/CD11b+ and CD14+/CD142+) are related to long-term prognosis for cardiovascular mortality in STEMI patients. *Int J Cardiol.* (2017) 227:876–81. 10.1016/j.ijcard.2016.11.302 27915085

[23] Sluijter JPG Davidson SM Boulanger CM Buzás EI De Kleijn DPV Engel FB Extracellular vesicles in diagnostics and therapy of the ischaemic heart: position Paper from the working group on cellular biology of the heart of the european society of cardiology. *Cardiovasc Res.* (2018) 114:19–34. 10.1093/cvr/cvx211 29106545 PMC5852624

[24] Suades R Padró T Crespo J Ramaiola I Martin-Yuste V Sabaté M Circulating microparticle signature in coronary and peripheral blood of ST elevation myocardial infarction patients in relation to pain-to-PCI elapsed time. *Int J Cardiol.* (2016) 202:378–87. 10.1016/j.ijcard.2015.09.011 26432487

[25] Chiva-Blanch G Suades R Crespo J Peña E Padró T Jiménez-Xarrié E Microparticle shedding from neural progenitor cells and vascular compartment cells is increased in ischemic stroke. *PLoS One.* (2016) 11:e0148176. 10.1371/journal.pone.0148176 26815842 PMC4729528

[26] Berezin AE Kremzer AA Martovitskaya YV Samura TA Berezina TA. The predictive role of circulating microparticles in patients with chronic heart failure. *BBA Clin.* (2015) 3:18–24. 10.1016/j.bbacli.2014.11.006 26672475 PMC4661507

[27] Berezin AE Kremzer AA Samura TA Berezina TA. Altered signature of apoptotic endothelial cell-derived microvesicles predicts chronic heart failure phenotypes. *Biomark Med.* (2019) 13:737–50. 10.2217/bmm-2018-0449 31157550

[28] Berezin AE Kremzer AA Martovitskaya YV Berezina TA Gromenko EA. Pattern of endothelial progenitor cells and apoptotic endothelial cell-derived microparticles in chronic heart failure patients with preserved and reduced left ventricular ejection fraction. *EBioMedicine.* (2016) 4:86–94. 10.1016/j.ebiom.2016.01.018 26981573 PMC4776070

[29] Nozaki T Sugiyama S Sugamura K Ohba K Matsuzawa Y Konishi M Prognostic value of endothelial microparticles in patients with heart failure. *Eur J Heart Fail.* (2010) 12:1223–8. 10.1093/eurjhf/hfq145 20817695

[30] Sionis A Suades R Sans-Roselló J Sánchez-Martínez M Crespo J Padró T Circulating microparticles are associated with clinical severity of persistent ST-segment elevation myocardial infarction complicated with cardiogenic shock. *Int J Cardiol.* (2018) 258:249–58. 10.1016/j.ijcard.2017.10.044 29544939

[31] Nieuwland R Berckmans RJ McGregor S Böing AN Romijn FPHTM Westendorp RGJ Cellular origin and procoagulant properties of microparticles in meningococcal sepsis. *Blood.* (2000) 95:930–5. 10.1182/blood.V95.3.930.003k46_930_935 10648405

[32] Inamdar AA Inamdar AC. Heart Failure: diagnosis, management and utilization. *J Clin Med.* (2016) 5:62. 10.3390/jcm5070062 27367736 PMC4961993

[33] Biasucci LM La Rosa G Pedicino D D’Aiello A Galli M Liuzzo G. Where does inflammation fit? *Curr Cardiol Rep.* (2017) 19:84. 10.1007/s11886-017-0896-0 28779286

[34] Chiva-Blanch G Padró T Alonso R Crespo J Perez De Isla L Mata P Liquid biopsy of extracellular microvesicles maps coronary calcification and atherosclerotic plaque in asymptomatic patients with familial hypercholesterolemia: a computed tomographic angiography imaging study. *Arterioscler Thromb Vasc Biol.* (2019) 39:945–55. 10.1161/ATVBAHA.118.312414 30866660

[35] Gkaliagkousi E Gavriilaki E Yiannaki E Vasileiadis I Nikolaidou B Lazaridis A Platelet microvesicles are associated with the severity of coronary artery disease: comparison between peripheral and coronary circulation. *J Thromb Thrombolysis.* (2021) 51:1138–43. 10.1007/s11239-020-02302-5 33043416

[36] Chiva-Blanch G Crespo J Suades R Arderiu G Padró T Vilahur G CD142+/CD61+, CD146+ and CD45+ microparticles predict cardiovascular events in high risk patients following a Mediterranean diet supplemented with nuts. *Thromb Haemost.* (2016) 116:103–14. 10.1160/TH16-02-0130 27052787

[37] Chiva-Blanch G Suades R Crespo J Vilahur G Arderiu G Padró T CD3+/CD45+ and SMA-α+ circulating microparticles are increased in individuals at high cardiovascular risk who will develop a major cardiovascular event. *Int J Cardiol.* (2016) 208:147–9. 10.1016/j.ijcard.2016.01.211 26859321

[38] Dick SA Epelman S. Chronic heart failure and inflammation. What do we really know? *Circ Res.* (2016) 119:159–76. 10.1161/CIRCRESAHA.116.308030 27340274

[39] Dutka M Bobiński R Ulman-Włodarz I Hajduga M Bujok J Pająk C Various aspects of inflammation in heart failure. *Heart Fail Rev.* (2020) 25:537–48. 10.1007/s10741-019-09875-1 31705352 PMC7181445

