# Supplementary material for: New factors in heart failure pathophysiology: Immunity cells release of extracellular vesicles
Source: Front Cardiovasc Med. 2022 Nov 3;9:939625. doi: 10.3389/fcvm.2022.939625 (PMC9669903; doi:10.3389/fcvm.2022.939625)
Supplement: Supplementary file 1 [file Data_Sheet_1.pdf]

New Factors in Heart Failure Pathophysiology: Immunity cells release of extracellular vesicles

Supplementary Material

Alba Vilella-Figuerola[1,2], Teresa Padró[1,3], Eulàlia Roig[4], Sònia Mirabet[3,4†], Lina Badimon[1,3,5†*]

[1]Cardiovascular-Program-ICCC, IR-Hospital Santa Creu i Sant Pau, IIB-Sant Pau, Barcelona, Spain

2Biochemistry and Molecular Biology Department, Universitat Autònoma de Barcelona (UAB), Barcelona, Spain

[3]Centro de Investigación Biomédica en Red Cardiovascular, Instituto de Salud Carlos III, Madrid, Spain

[4]Heart Failure Group, Cardiology Department, Hospital Santa Creu i Sant Pau, Barcelona, Spain

[5]UAB-Chair Cardiovascular Research, Barcelona, Spain

[†]These authors have contributed equally to this work.

1 Supplementary Data

Western blot analysis of EVs

Isolated EVs were lysed with M2 buffer (50mmol/L TRIS pH 7.5, 150mmol/L NaCl, 1% SDS, 1% Triton-X-100, protease inhibitor) and the obtained protein was precipitated overnight at -20ºC, with 7 volumes of cold acetone. In addition, protein from human myocardial tissue, human monocytes and human platelets (with residual white cell fraction) was extracted with RIPA buffer (50mmol/L TRIS-HCl pH 6.8, 150mmol/L NaCl, 1% NP-40, 0.5% sodium deoxycholate, 0.5% SDS, protease inhibitor, adjusted to pH 8). Protein in the extracts was quantified with the 2-D Quant Kit (GE Healthcare, Germany).

Protein (5µg) from EVs lysates and myocardial tissue, monocyte and platelet-white blood cells extracts were loaded into SDS-PAGE (8% and 15%) gels under denaturing (CD3, CD11b) and non-denaturing (CD14) conditions. Protein detection was pursued using the antibodies listed in **Supplemental Table 3** as previously described(1).

Chemiluminescent imaging analysis was performed in the ChemiDocTM XRS (Bio-Rad Laboratories Inc) using the Quantity-One (version 4.6.8, Bio-Rad Laboratories Inc) and the Image Lab™ (version 6.0.1, Bio-Rad Laboratories Inc) software.

2 Extended Results

Clinical characteristics of the study population

Compared to controls, a higher number of cHF patients had chronic kidney disease (38.6% vs 3.3%, p<0.001) and diabetes (44.5% vs 16%, p<0.001), while 53.7% of cHF presented dyslipidaemia compared to 75% of controls (p=0.004). Also, 68.9% of cHF (55% of controls), suffered from hypertension (p=0.067). Regarding medication, cHF were treated with angiotensin-inhibition related drugs, beta-blockers and diuretics. In addition, more cHF patients were under antiplatelet, anticoagulant and anti-diabetic treatment (**Table 1**).

Ischaemic disease was the major cause of cHF, affecting 36.1% of the patients. Other major causes were dilated cardiomyopathy (20.1%), hypertensive cardiomyopathy (15.9%), heart valve disease (14.2%) and hypertrophic cardiomyopathy (10%) (**Table 2**). As presented in **Table 2**, 19 cHF patients had adverse cardiovascular events during follow-up, including acute myocardial infarction (1.6%), stroke (6.7%), aortic dissection (0.8%), cardiogenic shock (0.8%) and heart transplantation (8.4%; 1 of the patients had a previous stroke). In addition, 5.8% of the patients suffered a cardiovascular death, while other-cause death occurred in 8.4% of the cases. cHF-related hospitalisation occurred in 47.8% of cases and 13.4% of patients were admitted to the emergency department during follow-up.

3 Supplementary Tables

Supplemental table 1: Patients' characteristics at inclusion, considering New York Heart Association classification

	NHYA II n=52	NYHA III-IV n=67	P-value
**DEMOGRAPHIC CHARACTERISTICS; mean±SD**			
Male/Female, n	37/15	44/23	0.525
Age, years	66.37±12.72	67.61±11.18	0.639
Systolic blood pressure, mmHg	123.31±19.73	118.28±18.57	0.160
Diastolic blood pressure, mmHg	74.54±11.47	73.45±10.87	0.599
Left ventricular ejection fraction, %	45.67±19.24	45.52±18.92	0.964
**RISK FACTORS; n (%)**			
Smokers	6 (11.5)	7 (10.4)	0.850
Hypertension	35 (67.3)	47 (70.1)	0.740
Pulmonary hypertension	15 (28.8)	34 (50.7)	**0.008**
Diabetes mellitus	22 (42.3)	31 (46.2)	0.666
Dyslipidaemia	27 (51.9)	37 (55.2)	0.720
Chronic kidney disease	18 (34.6)	28 (41.7)	0.526
Atrial fibrillation	20 (38.4)	30 (44.7)	0.545
**CLINICAL HISTORY; n (%)**			
**cHF aetiology**			0.761
Ischaemic	18 (34.6)	25 (37.3)	
Non-ischaemic	34 (65.4)	42 (62.7)	
Hospitalisations in the 6 months prior study initiation	18 (43.7)	31 (42.3)	0.209
**Re-events in the year prior study initiation**	3 (5.7)	6 (8.9)	0.514
Percutaneous coronary intervention	2 (66.6)	2 (33.4)	0.343
Coronary artery bypass grafting	1 (33.4)	1 (16.6)	0.571
Medical treatment	0 (0)	3 (50)	0.134
**BIOCHEMISTRY; mean±SD**			
Haemoglobin, mg/dl	132.98±19.59	127.45±17.50	0.096
Creatinine, mg/dl	1.31±0.59	1.36±0.49	0.197
C-Reactive Protein, mg/ml	6.57±8.73	8.44±12.46	0.345
NT-proBNP, pg/ml	2173.38±4316.14	3561.28±4057.0	**0.001**
High-sensitive troponin T, ng/l	22.78±14.59	31.64±24.18	**0.041**
Erythrocytes, 10^6/mm^3	4.1±0.59	3.88±0.82	0.103
Platelets, 10^3/mm^3	178.12±51.26	169.28±61.12	0.326
Leukocytes, mm^3	7421.76±2172.93	7429.57±1773.26	0.541
Neutrophils, 10^9/L	4.74±1.15	4.74±1.77	0.891
Monocytes, 10^9/L	0.69±0.26	0.8±0.66	0.950

cHF: chronic heart failure; **NT-proBNP**: N-terminal pro-hormone of brain natriuretic peptide; **NYHA**: New York Heart Association; **SD**: standard deviation

Supplemental table 2: Follow-up events and outcomes, considering NYHA classification

**MAJOR OUTCOMES DURING FOLLOW-UP; n (%)**	**NHYA II n=52**	**NYHA III-IV n=67**	**P-value**
**Cardiovascular event**[†]	12 (23.1)	14 (20.9)	0.775
Stroke	3 (5.7)	5 (7.4)	0.714
Aortic dissection	1 (1.9)	0 (0)	0.254
AMI + Cardiogenic shock	2 (3.8)	1 (1.4)	0.417
HTx/HTx waiting list	0 (0)/1 (1.9)	10 (14.9)/0 (0)	**0.004**/0.254
CV death	3 (5.7)	4 (5.9)	0.963
Emergency hospital admission for cHF	6 (11.5)	10 (14.9)	0.591
Rehospitalisation	21 (40.3)	36 (53.7)	0.148
Aortic aneurism	0 (0)	1 (1.4)	0.376
Other death causes[‡]	2 (3.8)	8 (11.9)	0.114

[†]Includes patients that suffered a stroke, an aortic dissection, an AMI, a cardiogenic shock, a CV death (mainly due to cHF) or were admitted to the emergency department. It does not include patients that underwent a HTx.

[‡]Includes patients that died due to a septic shock, a haemorrhage or a non-successful HTx.

AMI: acute myocardial infarction; **CV**: cardiovascular; **cHF**: chronic heart failure; **HTx**: heart transplantation

Supplemental table 3: Cell surface molecules for extracellular microvesicle identification and characterisation

ANTIBODIES FOR FLOW CYTOMETRY ANALYSIS									
	**EXPRESSION**	**MARKER**	**ALTERNATIVE NAME**	**CONJUGATION**	**CLONE**	**DILUTION**	**HOST**	**COMPANY**	**CATALOGUE NUMBER**
**Annexin V**	Widely expressed	PS^{+}	PS-binding protein	CF405	-	5µl (1:10)	-	Immunostep	ANXVCFB-200T
**Leukocyte markers**	Lymphocytes T	CD3^{+}	T-cell coreceptor	FITC	HIT3b	5µl (1:10)	Mouse	Immunotools	21810033
	Leukocytes	CD45^{+}	LCA	PE	MEM-28	5µl (1:10)	Mouse	Immunotools	21270454
	Neutrophils, monocytes	CD11b^{+}	MAC-1/α-M integrin	FITC	MEM-174	5µl (1:10)	Mouse	Immunotools	21279113
	Monocytes, macrophages	CD14^{+}	LPS-receptor	PE	M5E2	5µl (1:100)	Mouse	BD Pharmingen	555398
	Activated lymphocytes	CD29^{+}	ITGBI	FITC	HI29a	5µl (1:10)	Mouse	Immunotools	21810293
	Granulocytes, neutrophils	CD15^{+}	Sialyl Lewis X	PE	MEM-158	5µl (1:10)	Mouse	Immunotools	21270154
	Natural killers	CD56^{+}	NCAM1	FITC	B-A19	5µl (1:10)	Mouse	Immunotools	21810563
	Natural killers, monocytes	CD16^{+}	FcγRIII	FITC	HI16a	5µl (1:10)	Mouse	Immunotools	21810163

*At staining (final volume of reagents: 50µl [5µl of isolated EVs, 5µl of Annexin V, ~5µl of antibody-FITC, ~5µl of antibody-PE, adjust to final a volume of 50µl with annexin binding buffer]).
FITC: Fluorescein isothiocyanate; **PE**: Phycoerythrin; **PS**: Phosphatidylserine; **LCA**: Leukocyte common antigen; **MAC-1**: Macrophage-1 Antigen; **LPS**: Lipopolysaccharide; **ITGB1**: Integrin β-1; **NCAM1**: Neural cell adhesion molecule-1; **FcγRIII**: Receptor Fcγ III.

ANTIBODIES FOR WESTERN BLOT ANALYSIS								
	**EXPRESSION**	**MARKER**	**ALTERNATIVE NAME**	**CLONE**	**DILUTION**	**HOST**	**COMPANY**	**CATALOGUE NUMBER**
**Leukocyte markers**	Lymphocytes T	CD3	T-cell coreceptor	Polyclonal	1:2000	Rabbit	Abcam	ab16044
	Neutrophils, monocytes	CD11b	MAC-1/α-M integrin	EP1345Y	1:1000	Rabbit	Abcam	ab52478
	Monocytes, macrophages	CD14	LPS-receptor	4B4F12	1:500	Mouse	Abcam	ab182032

LCA: Leukocyte common antigen; **MAC-1**: Macrophage-1 Antigen

Supplemental table 4: Association between AV^+-EVs and biochemical parameters

	Leukocytes		Neutrophils		Monocytes	
	Spearman's Rho	P-value	Spearman's Rho	P-value	Spearman's Rho	P-value
**Cell-origin markers**						
$CD3^+$	0.041	0.664	0.118	0.451	-0.004	0.978
$CD45^+$	0.034	0.720	0.095	0.550	0.085	0.593
$CD3^+/CD45^+$	0.069	0.464	0.176	0.259	0.067	0.670
$CD14^+$	-0.057	0.543	0.101	0.517	0.062	0.695
$CD15^+$	0.025	0.789	0.222	0.152	-0.067	0.670
$CD56^+$	-0.033	0.727	-0.014	0.927	0.055	0.727
$CD16^+$	0.051	0.787	-0.248	0.354	-0.003	0.991
$CD16^+/CD14^+$	-0.244	0.202	-0.350	0.201	-0.163	0.561
**Activation markers**						
$CD11b^+$	0.037	0.695	0.146	0.349	-0.152	0.329
$CD11b^+/CD14^+$	-0.072	0.450	0.120	0.461	-0.101	0.535
$CD29^+$	0.033	0.724	-0.027	0.865	0.170	0.276
$CD29^+/CD15^+$	0.045	0.630	0.213	0.171	0.032	0.837

	C-Reactive Protein		NT-proBNP		High-sensitive troponin T	
	Spearman's Rho	P-value	Spearman's Rho	P-value	Spearman's Rho	P-value
**Cell-origin markers**						
$CD3^+$	-0.082	0.381	0.220	**0.016**	0.147	0.113
$CD45^+$	-0.118	0.212	-0.055	0.558	0.091	0.331
$CD3^+/CD45^+$	-0.096	0.311	0.252	**0.006**	0.217	**0.020**
$CD14^+$	-0.089	0.343	0.123	0.183	0.053	0.573
$CD15^+$	0.063	0.504	0.162	0.081	0.084	0.372
$CD56^+$	-0.024	0.800	0.082	0.378	-0.045	0.633
$CD16^+$	-0.086	0.646	0.034	0.853	0.098	0.599
$CD16^+/CD14^+$	-0.011	0.953	-0.031	0.867	-0.140	0.462
**Activation markers**						
$CD11b^+$	0.060	0.526	0.145	0.116	0.082	0.382
$CD11b^+/CD14^+$	-0.017	0.858	0.108	0.252	0.133	0.161
$CD29^+$	-0.059	0.527	0.028	0.765	0.038	0.687
$CD29^+/CD15^+$	0.040	0.668	0.175	0.057	0.162	0.082

AV^+: annexin V^+; **EVs**: extracellular vesicles; **NTproBNP**: N-terminal pro-hormone of brain natriuretic peptide

Supplemental table 5: Comparison of EVs levels according to disease severity and controls.

mean±SD	NYHA II	NYHA III-IV	CTLs	P-value NYHA II vs III-IV	P-value NYHA II vs CTLs	P-value NYHA III-IV vs CTLs
$CD3^{+}/AV^{+}$	2 [0-4]	4 [1.04-10]	0 [0-2]	**0.017**	**0.009**	**0.000**
$CD45^{+}/AV^{+}$	32 [16.55-44]	46 [26.6-64]	16 [8-34]	**0.011**	**0.003**	**0.000**
$CD3^{+}/CD45^{+}/AV^{+}$	0 [0-2]	2 [0-5.7]	0 [0-2]	**0.008**	0.100	**0.000**
$CD11b^{+}/AV^{+}$	9.75 [2.94-19.5]	17.1 [6-42]	4 [0-10]	**0.006**	**0.001**	**0.000**
$CD29^{+}/CD15^{+}/AV^{+}$	7.6 [2-18]	14 [3.8-32]	0 [0-2]	**0.048**	**0.000**	**0.000**

CTLs: controls; **NYHA**: New York Heart Association; **SD**: standard deviation

Supplemental table 6: ROC curve analysis for the discrimination of NYHA severity classification

	**AUC±SD (95% CI)**	**P-value**	**Sensitivity**	**Specificity**
CD11b$^+$/AV$^+$	0.648±0.050 (0.549-0.746)	**0.006**	0.627	0.615
CD45$^+$/AV$^+$	0.637±0.051 (0.536-0.738)	**0.011**	0.646	0.558
CD3$^+$/CD45$^+$/AV$^+$	0.636±0.051 (0.536-0.737)	**0.011**	0.615	0.596
CD3$^+$/AV$^+$	0.626±0.051 (0.526-0.726)	**0.019**	0.552	0.654
CD29$^+$/CD15$^+$/AV$^+$	0.606±0.052 (0.504-0.707)	**0.048**	0.597	0.577
Clustered CD11b$^+$/AV$^+$ and CD45$^+$/AV$^+$	0.658±0.050 (0.559-0.757)	**0.003**	0.662	0.615
Clustered CD11b$^+$/AV$^+$ and CD3$^+$/CD45$^+$/AV$^+$	0.635±0.051 (0.535-0.735)	**0.012**	0.585	0.596
NT-proBNP	0.676±0.050 (0.578-0.774)	**0.001**	0.672	0.655
High sensitivity Troponin T	0.611±0.052 (0.508-0.713)	**0.041**	0.606	0.529
Clustered CD11b$^+$/AV$^+$ and NT-proBNP	0.702±0.049 (0.607-0.797)	**0.000**	0.716	0.615
Clustered CD45$^+$/AV$^+$ and NT-proBNP	0.705±0.048 (0.610-0.799)	**0.000**	0.677	0.712
Clustered CD3$^+$/CD45$^+$/AV$^+$ with NT-proBNP	0.691±0.049 (0.596-0.787)	**0.000**	0.631	0.673
Clustered CD11b$^+$/AV$^+$, CD45$^+$/AV$^+$ and NT-proBNP	0.704±0.048 (0.609-0.798)	**0.000**	0.738	0.615
Clustered CD11b$^+$/AV$^+$, CD3$^+$/CD15$^+$/AV$^+$ and NT-proBNP	0.690±0.049 (0.594-0.786)	**0.000**	0.677	0.615

AUC: area under the curve; **CI**: confidence interval; **SD**: standard deviation; **NT-proBNP**: N-terminal pro-hormone of brain natriuretic peptide; **NYHA**: New York Heart Association

Supplemental table 7: Patient characteristics depending on ejection fraction classification

	HFpEF n=59	HFrEF n=60	P-value
**DEMOGRAPHIC CHARACTERISTICS; mean±SD**			
Male/Female, n	31/28	50/10	**0.000**
Age, years	71.1±9.6	63±12.5	**0.000**
Systolic blood pressure, mmHg	128.3±16.6	112.7±18.3	**0.000**
Diastolic blood pressure, mmHg	76.3±8.9	71.5±12.5	**0.007**
Left ventricular ejection fraction, %	63±9	28.47±6.2	**0.000**
**RISK FACTORS; n (%)**			
Smokers	7 (11.8)	6 (10)	0.744
Hypertension	48 (81.3)	34 (56.6)	**0.004**
Pulmonary hypertension	22 (37.2)	27 (45)	0.268
Diabetes mellitus	23 (38.9)	30 (50)	0.227
Dyslipidaemia	35 (59.3)	29 (48.3)	0.229
Chronic kidney disease	26 (44)	20 (33.3)	0.289
Atrial fibrillation	31 (52.5)	19 (31.6)	**0.025**
**BACKGROUND MEDICATION; n (%)**			
Angiotensin-converting-enzyme inhibitors	22 (37.2)	26 (43.3)	0.502
Angiotensin II receptor blockers	23 (38.9)	12 (20)	**0.023**
Beta-blockers	40 (67.7)	60 (100)	**0.000**
Aldosterone antagonists	17 (28.8)	49 (81.6)	**0.000**
Diuretics†	52 (88.1)	52 (86.6)	0.809
Angiotensin receptor neprilysin inhibitors	0 (0)	17 (28.3)	**0.000**
Ivabradine	2 (3.3)	12 (20)	**0.005**
Statins	36 (61)	41 (68.3)	0.404
Insulin	5 (8.4)	11 (18.3)	0.115
Anti-diabetic drugs	20 (33.8)	20 (33.3)	0.948
Antiplatelet agents	14 (23.7)	32 (53.3)	**0.001**
Anticoagulants	34 (57.6)	27 (45)	0.148
Anti-arrhythmic drugs	13 (22)	13 (21.6)	0.961

†Includes: furosemide, hydrochlorothiazide, torasemide and indapamide.

HFpEF: heart failure with preserved ejection fraction; **HFrEF**: heart failure with reduced ejection fraction; **SD**: standard deviation

Supplemental table 8: Clinical characteristics of patients at baseline

	HFpEF n=59	HFrEF n=60	P-value
**CLINICAL HISTORY; n (%)**			
**cHF aetiology**			
Ischaemic	14 (23.7)	29 (48.3)	**0.005**
Non-ischaemic	45 (76.2)	31 (51.6)	
Hypertensive cardiomyopathy	16 (27.1)	3 (5)	**0.001**
Dilated cardiomyopathy	0 (0)	24 (40)	**0.000**
Hypertrophic cardiomyopathy	12 (20)	0 (0)	**0.001**
Heart valve disease	13 (22)	4 (6.6)	**0.017**
Other	4 (6.7)	0 (0)	**0.040**
**New York Heart Association cHF stage**			
NYHA I	0 (0)	0 (0)	1.000
NYHA II	25 (42.3)	27 (45)	0.773
NYHA III	34 (57.6)	32 (53.3)	0.637
NYHA IV	0 (0)	1 (1.6)	0.319
Hospitalisations in the 6 months prior study initiation	26 (44)	23 (38.3)	0.420
**Re-events in the year prior study initiation**	2 (3.3)	7 (11.6)	0.088
Percutaneous coronary intervention	0 (0)	4 (57.1)	0.151
Coronary artery bypass grafting	1 (50)	1 (14.3)	0.284
Medical treatment	1 (50)	2 (28.6)	0.571
**BIOCHEMISTRY; mean±SD**			
Haemoglobin, mg/dl	126.2±17.7	133.4±18.7	0.060
Creatinine, mg/dl	1.39±0.58	1.29±0.49	0.427
C-Reactive Protein, mg/ml	9.2±13.9	6.1±6.8	0.185
NT-proBNP, pg/ml	2782.6±4712.2	3124.1±3685.5	0.113
High-sensitive troponin T, ng/l	26.6±22.1	28.9±19.8	0.371
Erythrocytes, 10^6/mm^3	3.86±0.64	4.1±0.81	0.052
Platelets, 10^3/mm^3	168.9±49.6	177.4±63.4	0.462
Leukocytes, mm^3	7191±1744.8	7661.03±2124.9	0.377
Neutrophils, 10^9/L	4.39±1.09	5.03±1.79	0.133
Monocytes, 10^9/L	0.67±0.2	0.82±0.69	0.651

HFpEF: heart failure with preserved ejection fraction; **HFrEF**: heart failure with reduced ejection fraction; **NT-proBNP**: N-terminal pro-hormone of brain natriuretic peptide; **NYHA**: New York Heart Association; **SD**: standard deviation

Supplemental table 9: Follow-up outcome

MAJOR OUTCOMES DURING FOLLOW-UP; n (%)	HFpEF n=59	HFrEF n=60	P-value
**Cardiovascular event**[†]	11 (18.6)	15 (25)	0.402
Stroke	3 (5)	5 (8.3)	0.479
Aortic dissection	1 (1.6)	0 (0)	0.311
AMI + Cardiogenic shock	1 (1.6)	2 (3.3)	0.569
HTx/HTx waiting list	3 (5)/0 (0)	7 (11.6)/1 (1.6)	0.196/0.319
CV death	3 (5)	4 (6.6)	0.714
Emergency hospital admission for cHF	7 (11.8)	9 (15)	0.616
Rehospitalisation	29 (49.1)	28 (46.6)	0.786
Aortic aneurism	1 (1.6)	0 (0)	0.311
Other death causes[‡]	7 (11.8)	3 (5)	0.177

[†]Includes patients that suffered a stroke, an aortic dissection, an AMI, a cardiogenic shock, a CV death (mainly due to cHF) or were admitted to the emergency department. It does not include patients that underwent a HTx.

[‡]Includes patients that died due to a septic shock, a haemorrhage or a non-successful HTx.

AMI: acute myocardial infarction; **CV**: cardiovascular; **HFpEF**: heart failure with preserved ejection fraction; **HFrEF**: heart failure with reduced ejection fraction; **HTx**: heart transplantation

Supplemental table 10: ROC curve analysis for the discrimination of NYHA severity classification in ischaemic patients.

	AUC±SD (95% CI)	P-value	Sensitivity	Specificity
$CD11b^+/AV^+$	0.848±0.058 (0.734-0.961)	**0.000**	0.800	0.722
$CD29^+/CD15^+/AV^+$	0.811±0.065 (0.683-0.939)	**0.001**	0.760	0.722
$CD3^+/AV^+$	0.723±0.077 (0.573-0.873)	**0.013**	0.640	0.556
$CD45^+/AV^+$	0.714±0.081 (0.555-0.874)	**0.019**	0.667	0.778
$CD3^+/CD45^+/AV^+$	0.700±0.083 (0.537-0.864)	**0.029**	0.652	0.667
$CD11b^+/CD14^+/AV^+$	0.661±0.085 (0.493-0.828)	0.081	0.522	0.778
Clustered $CD11b^+/AV^+$ and $CD29^+/CD15^+/AV^+$	0.858±0.056 (0.747-0.968)	**0.000**	0.800	0.833
Clustered $CD11b^+/AV^+$ and $CD3^+/AV^+$	0.857±0.057 (0.746-0.968)	**0.000**	0.760	0.833
Clustered $CD29^+/CD15^+/AV^+$ and $CD3^+/AV^+$	0.812±0.065 (0.686-0.939)	**0.001**	0.760	0.722
NT-proBNP	0.816±0.071 (0.676-0.955)	**0.000**	0.800	0.722
High sensitivity Troponin T	0.640±0.082 (0.472-0.808)	0.127	0.600	0.588
Clustered $CD11b^+/AV^+$ and NT-proBNP	0.849±0.057 (0.737-0.961)	**0.000**	0.760	0.778
Clustered $CD29^+/CD15^+/AV^+$ with NT-proBNP	0.809±0.065 (0.681-0.937)	**0.001**	0.800	0.667
Clustered $CD3^+/AV^+$ and NT-proBNP	0.700±0.079 (0.545-0.855)	**0.027**	0.680	0.556
Clustered $CD11b^+/AV^+$, $CD29^+/CD45^+/AV^+$ and NT-proBNP	0.858±0.056 (0.747-0.968)	**0.000**	0.800	0.833
Clustered $CD11b^+/AV^+$, $CD3^+/AV^+$ and NT-proBNP	0.853±0.057 (0.741-0.965)	**0.000**	0.760	0.833

AUC: area under the curve; **CI**: confidence interval; **SD**: standard deviation; **NT-proBNP**: N-terminal pro-hormone of brain natriuretic peptide; **NYHA**: New York Heart Association

Supplemental table 11: EVs levels according myocardial infarction elapsed time in ischaemic cHF.

median [IQR]	AMI >1year before sample acquisition	AMI <1year before sample acquisition	P-value
$CD3^+/AV^+$	4.8 [1-13.8]	4 [1.47-13.42]	0.939
$CD45^+/AV^+$	45.8 [38.8-140]	38 [22-58]	0.337
$CD3^+/CD45^+/AV^+$	3.85 [1-5.85]	1.95 [0-6]	0.741
$CD11b^+/AV^+$	17.55 [15.55-24]	12 [6-40.92]	0.546
$CD14^+/AV^+$	2 [0-5.8]	3.8 [1.9-8]	0.335
$CD11b^+/CD14^+/AV^+$	0 [0-0]	0 [0-2]	0.093
$CD29^+/AV^+$	54 [31-91.75]	70.56 [44-135.45]	0.378
$CD15^+/AV^+$	36.6 [18-58.6]	26.49 [2-84]	0.755
$CD29^+/CD15^+/AV^+$	20 [11-32.05]	16.55 [2-37]	0.781
$CD56^+/AV^+$	4.85 [3-9.85]	8.75 [3.3-17.55]	0.378
$CD16^+/AV^+$	28 [28-28]	4.68 [4-15.56]	0.170
$CD16^+/CD14^+/AV^+$	0 [0-0]	0 [0-0]	0.684

AMI: acute myocardial infarction; **IQR**: interquartile range

3.1 Supplementary Figures

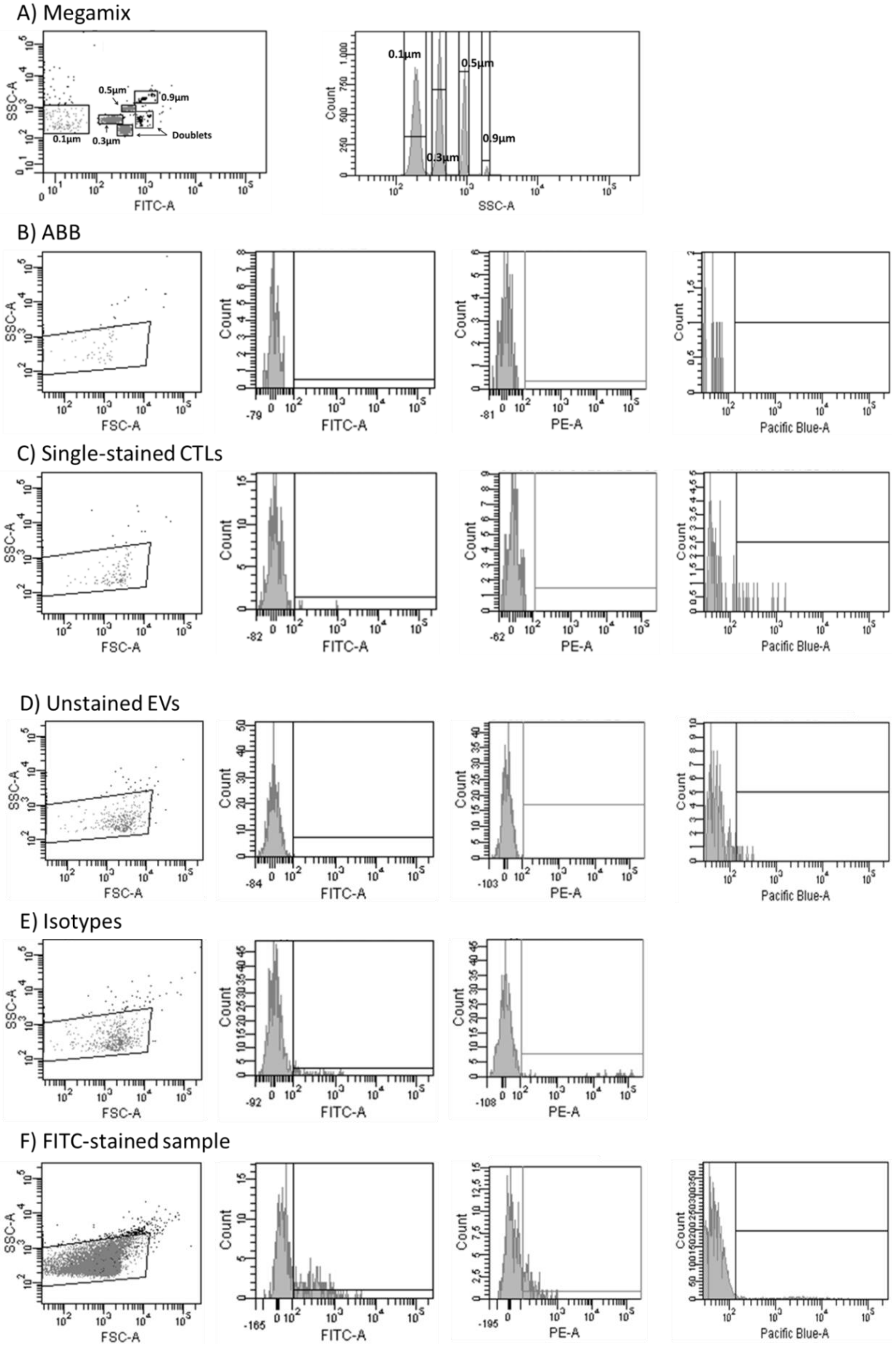

G) PE-stained sample

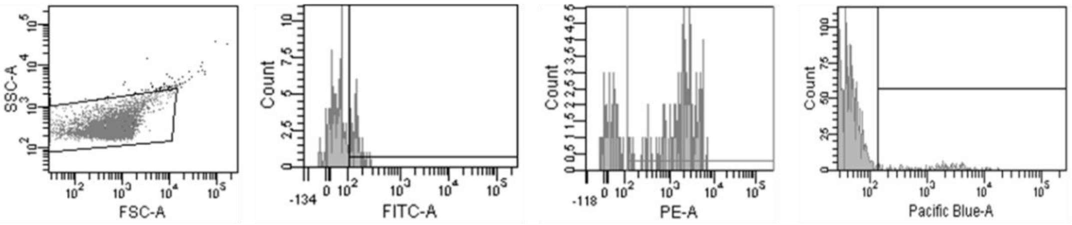

H) CFBlue-stained sample

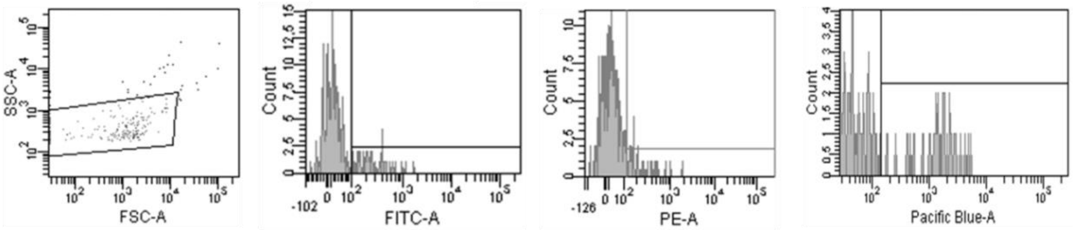

I) Dilution 1/4 of sample

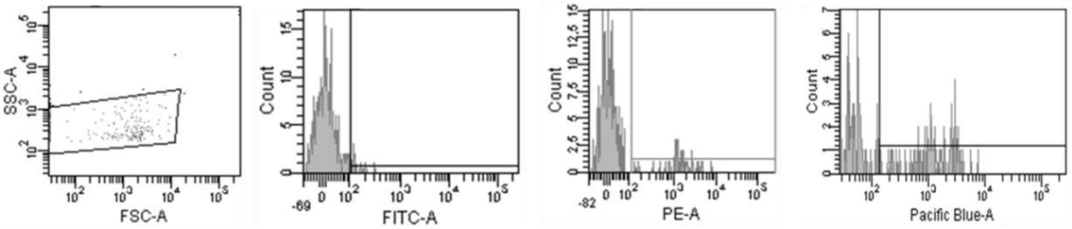

J) Dilution 1/10 of sample

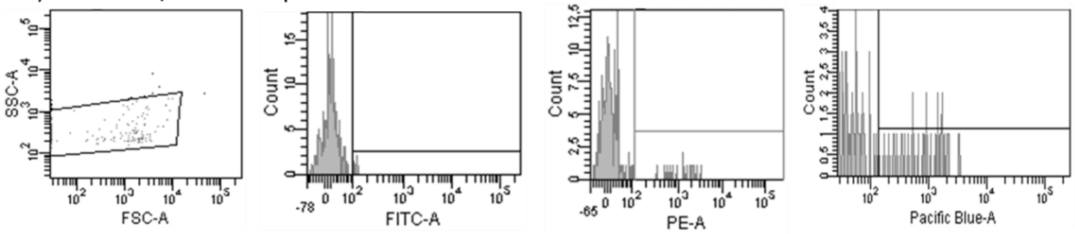

K) Dilution 1/50 of sample

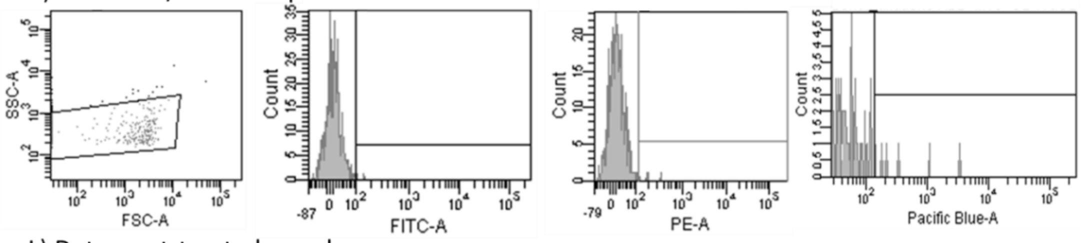

L) Detergent-treated sample

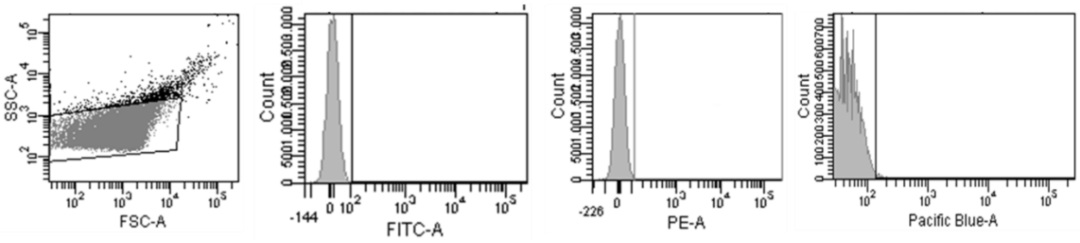

Supplemental Figure 1: **Flow cytometry controls for EVs analysis**. Representative FSC/SSC dot plots, and CFBlue (in the Pacific Blue channel), FITC and PE histograms of: **(A)** Megamix-Plus FSC beads; **(B)** Annexin Binding Buffer (ABB) only; **(C)** ABB with FITC-, PE-labelled antibodies and FCBlue-labelled Annexin V; **(D)** Unstained EVs; **(E)** FITC- and PE-labelled isotype controls; **(F)** FITC-stained sample; **(G)** PE-stained sample; **(H)** CFBlue-stained sample; **(I)** Dilution 1/4 of sample; **(J)** Dilution of 1/10 of sample; **(K)** Dilution of 1/50 of sample; **(L)** Detergent treated EVs sample (saponin 5% treatment in EVs-labelled with a FITC- and PE-antibody, as well as with FCBlue-labelled Annexin V). Note that the scale of the x axis in Pacific Blue histograms differs slightly from the x axis of FITC and PE histograms.

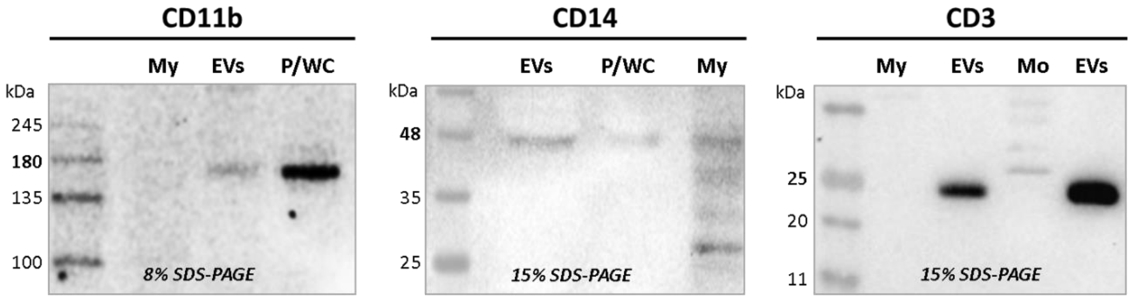

Supplemental Figure 2: **SDS-PAGE analysis to validate the markers determined by flow cytometry.** CD11b is expressed on the surface of many leukocytes (including monocytes, neutrophils, natural killer cells, granulocytes and macrophages) and it is found in P/WC extracts containing white cells and also in the EVs isolated from cHF patients' blood, but not in our control myocardium; CD14 (produced mostly by macrophages as part of the innate immune system) is found in the EVs, in less amount in the myocardium and in the control extract; CD3 (protein complex and T cell co-receptor that is involved in activating both cytotoxic T cell and T helper cells) is found in the EVs and not in the myocardial tissue or in monocytes.

EVs: extracellular microvesicles; **Mo**: monocytes; **My**: myocardium; **P/WC**: platelet-white blood cells extract

4 MiFlowCyt-EV: Author checklist

FRAMEWORK CRITERIA	COMPLETED CRITERIA
**1.1 Preanalytical variables conforming to MISEV guidelines**	Blood was collected from 119 chronic heart failure (cHF) individuals and 60 controls in 5ml 0.109M citrated plastic tubes (BD Vacutainer, Becton Dickinson) via antecubital vein puncture using a 20-gauge needle. Tubes were transported vertically at room temperature (RT).<br>Within 2 hours of blood withdrawal; platelet-depleted plasma was prepared by centrifugation (Eppendorf 5810R GLOOB04932 centrifuge, A-4-81 rotor, Eppendorf) at 1,560g for 20 minutes at 20ºC. A second centrifugation was then pursued (Eppendorf 5415R centrifuge, FA45-24-11 rotor, Eppendorf) at 1,500g for 10 minutes at 20ºC. The first centrifugation step was done with 4.5mL whole blood in the 5mL 0.109M citrated plastic tubes (BD Vacutainer, Becton Dickinson). Supernatant was collected 1mm above the buffy coat. The second centrifugation step was pursued with 650µl platelet-depleted plasma in 1.5mL Eppendorf tubes (Thermo Fisher Scientific). 300µl x2 aliquots of platelet-depleted plasma were transferred to 1.5mL low-protein binding Eppendorf tubes (Thermo Fisher Scientific) and snap-frozen in liquid nitrogen before being stored at −80 °C.
**1.2 Experimental design according to MIFlowCyt guidelines**	**1.1 Aim**: To compare the concentration of EVs from different parental origin in platelet-free plasma between individuals with cHF and age-, sex- and risk factor-matched controls without cHF. We hypothesise that the concentration of immune-derived EVs will be increased in individuals with cHF, as cHF has been associated with chronic and non-resolving inflammation.<br>**1.2 Keywords**: EVs; extracellular microvesicles, cHF; chronic heart failure; innate and immune cells, inflammation<br>**1.3 Experimental variables**: EVs from 119 individuals with cHF and 60 non-cHF controls. There was no significant difference in age, sex or smoking-status between individuals with and without cHF. Scatter-based triggering was used for the detection of particles.
**2.1 Sample staining details**	The presence of EVs was determined using parental-cell specific antibody staining. 5µl of EVs suspension was diluted in 30µl of filtered PBS (through 0.22µm pores) containing 2.5mM of $CaCl_2$ (Annexin Binding Buffer [ABB], BD Biosciences, San José) and stained with 5µl of a FITC-conjugated antibody, 5µl of a PE-conjugated antibody and 5µl of CFBlue-conjugated annexin V (please see **Supplemental Table 3** for an overview of the antibodies/reagents and concentrations used) for 20 minutes at 20ºC and protected from light. Matched isotype controls, were incubated in the same conditions and concentration as the labelling antibodies.
**2.2 Sample washing details**	EVs isolated by a two-step centrifugation were resuspended in PBS-0.32% citrated buffer, previously filtrated in 0.22µm-pore filters. The isolation was pursued by centrifuging (Eppendorf 5417R centrifuge, FA45-24-11 rotor, Eppendorf) the samples at 20,000g for 30 minutes at 20ºC, washing the pellet with PBS-0.32% citrated buffer and re-centrifuging the samples at 20,000g for 30 minutes at 20ºC. Supernatant was discarded and the pellet with EVs was resuspended to a total volume of 100µl of filtered PBS-0.32% citrated buffer.

FRAMEWORK CRITERIA	COMPLETED CRITERIA
	Thus, EV samples were washed before staining. Antibody labelling of EVs was stopped by diluting ten times the sample with buffer prior to FACS acquisition.
**2.3 Sample dilution details**	5µL of EV suspension was added to 15µL of reagents and 35µL of ABB (filtered PBS containing 2.5mM of $CaCl_2$), resulting in a 10-fold dilution. This 10-fold dilution was then diluted 10 times, with 50µL of sample added to 450µL of filtered ABB. EV concentration was semi-quantitatively estimated according to Nieuwland's formula(2) based on sample's volume (taking into account these dilutions), flow cytometer's flow rate and the number of fluorescence-positive events as described in **Methods** section.
**3.1 Buffer alone controls**	A buffer-only control of 0.22µm-filtered ABB (PBS containing 2.5mM of $CaCl_2$) was recorded at the same flow cytometer, with identical acquisition settings as all other samples, including triggering threshold, voltages, flow rate and acquisition time.
**3.2 Buffer with reagent controls**	A buffer with reagent control mimicking each of the used antibodies concentrations was recorded at the same flow cytometer and acquisition settings as all other samples, including triggering threshold, voltages, flow rate and acquisition time.
**3.3 Unstained controls**	Unstained controls were measured at the same dilution, as matched stained and isotype controls. Flow cytometer acquisition settings were maintained for all samples, including triggering threshold, voltages, flow rate and acquisition time.
**3.4 Isotype controls**	Isotype controls were used at the same concentration as matched stained controls and were recorded at the same dilution as matched stained and unstained controls and stained samples.<br>Flow cytometer acquisition settings were maintained for all samples, including triggering threshold, voltages, flow rate and acquisition time.
**3.5 Single-stained controls**	Single-stained controls of all used antibodies/CFBlue-conjugated annexin (see **Supplemental Table 3**) were analysed to aid compensation of used antibodies in the FITC, PE and Pacific Blue channels when excited by the 488nm laser.
**3.7 Serial dilutions**	Samples were serially diluted 4 times, with the sample diluted 1/4, 1/10 and 1/50 in 0.22µm-filtered ABB.
**3.8. Detergent treated EV-samples**	Stained samples, diluted 100-fold, were treated with 5% saponin for 20 min at 20°C to test the lability of the used antibodies and annexin V.
**4.1 Trigger Channel(s) and Threshold(s)**	Based on the buffer alone control, detection was triggered on the 488nm laser excited FITC channel and the 405nm laser excited by Pacific Blue, determined using CTS beads (Becton Dickinson, Franklin Lakes, NJ, USA).
**4.2 Flow Rate / Volumetric quantification**	Flow rate was calculated by the mass discharge method and considered constant throughout the analysis (~20µL/min). The FACSCantoII "low flow" rate mode was used in all analyses.
**4.3 Fluorescence Calibration**	NA<br>Cytometer CST beads (Becton Dickinson, Franklin Lakes, NJ, USA) controls to check cytometer performance were analysed each day. Megamix Plus FSC beads (BioCytex, Marseille, France), were also run to determine EVs size gates.<br>Nevertheless, because our analysis only required a positive/negative epitope discrimination, standardisation of the strength/intensity of florescence was not

FRAMEWORK CRITERIA	COMPLETED CRITERIA
	implemented in this study.
**4.4 Light Scatter Calibration**	Cytometer CSTs (Becton Dickinson, Franklin Lakes, NJ, USA) controls to check flow cytometer performance were analysed each working day. Megamix Plus FSC beads (BioCytex, Marseille, France) were also run to determine and establish EVs size gate.
**5.1 EV diameter/surface area/volume approximation**	NA
**5.2 EV refractive index approximation**	NA
**5.3 EV epitope number approximation**	NA
**6.1 Completion of MIFlowCyt checklist**	Completed MIFlowCyt checklist file has been included.
**6.2 Calibrated channel detection range**	NA
**6.3 EV number/concentration**	EVs number was calculated following Nieuwland's formula(2), which takes into consideration sample's volume, flow cytometer's flow rate and the number of fluorescence-positive events (N), as follows: EVs/µl = N x (Vf/Va) x (Vt/FR) x (1/Vi), where Vf(µl)=final volume of washed EVs suspension, Va(µl)=volume of washed EVs suspension used for each labelling analysis, Vt(µl)=volume of EVs suspension before fluorescence-activated cell sorting analysis, FR(µl/min)=flow rate of the cytometer at low mode (the average volume of EVs suspension analysed in 1min), 1 is the µl unit of volume, and Vi(µl)=original volume of plasma used for EVs isolation.
**6.4 EV brightness**	NA

5 MiFlowCyt: Compliant checklist

REQUIREMENT	COMPLETED CRITERIA
**1.1. Purpose**	To compare the concentration of EVs from different parental origin in platelet-free plasma between individuals with cHF and age-, sex- and risk factor-matched controls without cHF. We hypothesise that the concentration of immune-derived EVs will be increased in individuals with cHF, as cHF has been associated with chronic and non-resolving inflammation.
**1.2. Keywords**	Extracellular microvesicles, chronic heart failure; immune cells, inflammation
**1.3. Experiment variables**	EVs from 119 individuals with cHF and 60 non-cHF controls. There was no significant difference in age, sex or smoking-status between individuals with and without cHF. Scatter-based triggering was used for the detection of particles.
**1.4. Organization name and address**	Cardiovascular Program-ICCC<br>Institut de Recerca de l'Hospital de la Santa Creu i Sant Pau<br>Sant Antoni Maria Claret 167<br>08025 Barcelona<br>Spain
**1.5. Primary contact name and email address**	Prof. Lina Badimon<br>lbadimon@santpau.cat
**1.6. Date or time period of experiment**	10/01/2018 – 12/09/2019
**1.7. Conclusions**	cHF patients present increased levels of immune-derived EVs, which in addition correlate with severity, compared to non-cHF controls.
**1.8. Quality control measures**	Implemented quality control measures were cytometer CST beads (Becton Dickinson, Franklin Lakes, NJ, USA), which were run every working day to verify flow cytometer performance. Megamix Plus FSC beads (BioCytex, Marseille, France), were run each working day to determine EVs size gates.
**2.1.1.1. Sample description**	Washed EVs isolated from platelet-free plasma of cHF and non-cHF individuals.
**2.1.1.2. Biological sample source description**	Platelet-free plasma of cHF and non-cHF individuals.
**2.1.1.3. Biological sample source organism description**	Human
**2.1.2.2. Environmental sample location**	NA
**2.3. Sample treatment description**	EVs isolated by a two-step centrifugation were resuspended in PBS-0.32% citrated buffer, previously filtrated in 0.22µm-pore filters. The isolation was pursued by centrifuging (Eppendorf 5417R centrifuge, FA45-24-11 rotor, Eppendorf) the samples at 20,000g

REQUIREMENT	COMPLETED CRITERIA
	for 30 minutes at 20ºC, washing the pellet with PBS-0.32% citrated buffer and re-centrifuging the samples at 20,000g for 30 minutes at 20ºC. Supernatant was discarded and the pellet with EVs was resuspended to a total volume of 100µl of filtered PBS-0.32% citrated buffer.
**2.4. Fluorescence reagent(s) description**	EVs were triple-labelled with a combination of 3 fluorochromes, two corresponding to FITC- and PE-conjugated antibodies and the third one to FCBlue-conjugated annexin V, respectively. Used reagents are listed and characterised in **Supplemental Table 3**.
**3.1. Instrument manufacturer**	Becton Dickinson
**3.2. Instrument model**	FACSCantoII™
**3.3. Instrument configuration and settings**	EVs gating was optimised for detection as previously described(3–5). Additional information is included in this **Supplementary material**.
**4.1. List-mode data files**	NA<br>Despite data has not been uploaded to a public repository, it will be available upon request to the authors.
**4.2. Compensation description**	Single-stained controls of all used antibodies/CFBlue-conjugated annexin (see **Supplemental Table 3**) were analysed to aid compensation of used antibodies in the FITC, PE and Pacific Blue channels when excited by the 488nm laser.
**4.3. Data transformation details**	NA<br>Data transformation was not applied. Data from .cfs files were analysed by statistical software (SPSS version 26.0, IBM Corp. Armonk, NY).
**4.4.1. Gate description**	How gates were established and specific examples can be found in **Supplemental Figure 1**.
**4.4.2. Gate statistics**	Plots show the total number of events recorded for 1 minute at “low mode” flow rate.
**4.4.3. Gate boundaries**	More information and images can be found in **Supplemental Figure 1**.

6 Supplementary references

1. Lugano R, Peña E, Badimon L, Padró T. Aggregated low-density lipoprotein induce impairment of the cytoskeleton dynamics through urokinase-type plasminogen activator/urokinase-type plasminogen activator receptor in human vascular smooth muscle cell. J Thromb Haemost. 2012 Oct;10(10):2158–67.

2. Nieuwland R, Berckmans RJ, McGregor S, Böing AN, Romijn FPHTM, Westendorp RGJ, et al. Cellular origin and procoagulant properties of microparticles in meningococcal sepsis. Blood. 2000 Feb 1;95(3):930–5.

3. Suades R, Padró T, Vilahur G, Badimon L. Circulating and platelet-derived microparticles in human blood enhance thrombosis on atherosclerotic plaques. Thromb Haemost. 2012;108(6):1208–19.

4. Suades R, Padró T, Crespo J, Ramaiola I, Martin-Yuste V, Sabaté M, et al. Circulating microparticle signature in coronary and peripheral blood of ST elevation myocardial infarction patients in relation to pain-to-PCI elapsed time. Int J Cardiol. 2016 Jan 1;202:378–87.

5. Sionis A, Suades R, Sans-Roselló J, Sánchez-Martínez M, Crespo J, Padró T, et al. Circulating microparticles are associated with clinical severity of persistent ST-segment elevation myocardial infarction complicated with cardiogenic shock. Int J Cardiol. 2018 May 1;258:249–58.
